# Neural stemness contributes to cell tumorigenicity

**DOI:** 10.1186/s13578-021-00531-6

**Published:** 2021-01-19

**Authors:** Liyang Xu, Min Zhang, Lihua Shi, Xiaoli Yang, Lu Chen, Ning Cao, Anhua Lei, Ying Cao

**Affiliations:** MOE Key Laboratory of Model Animals for Disease Study, and Model Animal Research Center of the Medical School, Nanjing University, 12 Xuefu Road, Pukou High-Tech Zone, Nanjing, 210061 China

**Keywords:** Neural stem/progenitor cell, Neural ground state, Neural stemness, Tumor-initiating cell, Tumorigenicity, Tumorigenesis, Differentiation potential, Evo-devo

## Abstract

**Background:**

Previous studies demonstrated the dependence of cancer on nerve. Recently, a growing number of studies reveal that cancer cells share the property and regulatory network with neural stem/progenitor cells. However, relationship between the property of neural stemness and cell tumorigenicity is unknown.

**Results:**

We show that neural stem/progenitor cells, but not non-neural embryonic or somatic stem/progenitor cell types, exhibit tumorigenicity and the potential for differentiation into tissue types of all germ layers when they are placed in non-native environment by transplantation into immunodeficient nude mice. Likewise, cancer cells capable of tumor initiation have the property of neural stemness because of their abilities in neurosphere formation in neural stem cell-specific serum-free medium and in differentiation potential, in addition to their neuronal differentiation potential that was characterized previously. Moreover, loss of a pro-differentiation factor in myoblasts, which have no tumorigenicity, lead to the loss of myoblast identity, and gain of the property of neural stemness, tumorigenicity and potential for re-differentiation. By contrast, loss of neural stemness via differentiation results in the loss of tumorigenicity. These suggest that the property of neural stemness contributes to cell tumorigenicity, and tumor phenotypic heterogeneity might be an effect of differentiation potential of neural stemness. Bioinformatic analysis reveals that neural genes in general are correlated with embryonic development and cancer, in addition to their role in neural development; whereas non-neural genes are not. Most of neural specific genes emerged in typical species representing transition from unicellularity to multicellularity during evolution. Genes in *Monosiga brevicollis*, a unicellular species that is a closest known relative of metazoans, are biased toward neural cells.

**Conclusions:**

We suggest that the property of neural stemness is the source of cell tumorigenicity. This is due to that neural biased unicellular state is the ground state for multicellularity and hence cell type diversification or differentiation during evolution, and tumorigenesis is a process of restoration of neural ground state in somatic cells along a default route that is pre-determined by an evolutionary advantage of neural state.

## Introduction

Cancer is a complex disease characterized by inter- and intra-tumor heterogeneity and regulatory mechanisms that include thousands of cancer related genes, tens of millions of cancer related mutations, and numerous intertwined signaling pathways. There is not a unified explanation for cancer due to the complexity. In recent years, a growing number of studies revealed that cancer cells share properties of neural cells. Dependence of cancer on nerve has been observed since 1946 [1]. Hence, denervation was shown to suppress tumorigenesis [2, 3]. This dependence has been considered as a crosstalk between cancer and nerve [1]. However, cancer cells express elevated level of neurotrophic growth factors/receptors to promote cancer progression, and high expression of neural genes predicts higher aggressiveness [4–7]. Many cancers show a higher density of nerve or nerve development [3, 4, 8]. Besides, colorectal and gastric cancer stem cells show neuronal differentiation potential [9]. These studies suggest that neural genes and neural properties might play a cell-autonomous role, rather than a mere crosstalk, in promoting tumorigenesis.

We showed previously that cells of different cancer types were characteristic of neural stem/progenitor cells (NSCs/NPCs) because (1) cancer cells exhibit neuronal differentiation potential when endogenous cancer-promoting factors are inhibited, and (2) expression of most (if not all) cancer promoting genes or genes being upregulated/activated in cancer cells is neural specific or at least enriched in embryonic neural cells [10, 11]. In contrast, a substantial part of cancer repressor genes or the genes downregulated/silenced in cancer cells is expressed in non-neural cells or is not expressed in embryos [10]. Major genes/factors that regulate cancer cell malignant features are also expressed specifically in embryonic neural cells and/or play critical roles in neural development [10–12]. The similarity in regulatory network is not found between cancer cells and other types of cells, e.g., the mesenchymal cells. Therefore, tumorigenesis might represent a process of gradual loss of original cell identity and gain of properties of NSCs/NPCs [12]. This matches well with evolutionary studies, which suggest that cancer initiation and progression might reflect a reverse evolution from multicellularity to unicellularity [13, 14], or in other words, a convergence of different cell types to a unicellular state. This convergence should be at least partially due to the silencing/repression of tissue-specific or differentiation genes, leading to the loss of original cell features and to a dedifferentiation effect, and gain of stemness in cancer cells [15–17]. Recently, NPCs were shown to promote tumor growth and metastasis [18], and depletion of a gene in intestinal stem cells causes a switch to NSC-like state that subsequently drives tumorigenesis [19]. However, whether the property of NSCs/NPCs contributes to or is the source of cell tumorigenicity needs validation.

Among embryonic and adult cell types, ESCs and the induced pluripotent stem cells (iPS) are known to be tumorigenic [20]. However, tumorigenicity of NSCs derived from ESCs or iPS was reported, even though the derived cells were purified [21, 22]. Tumor formation derived from transplanted NSCs in a patient was also observed in a clinical practice [23]. Explanation for these observations remains unknown. ESCs are very close to primitive NSCs because the default fate of embryonic pluripotent cells, including amphibian blastula ectoderm cells and mammalian ESCs, is neural [24–27]. Considering together the embryonic neural property of cancer cells, all the studies suggest an intrinsic tumorigenic property of NSCs/NPCs. Here, we examined systematically the tumorigenicity of NSCs/NPCs and the similarity between cancer cells and NSCs/NPCs. We explored the effect of loss of a pro-differentiation factor on the identity of somatic cells and the gain of NSC fate and tumorigenicity. We also analyzed systematically conservativeness and correlation of neural genes with cancer. We conclude that the property of neural stemness is the source of cell tumorigenicity, and tumorigenesis is a process of restoration of neural ground state in somatic cells along a default route predetermined by evolution.

## Materials and methods

### Cell culture

C57BL/6 mESCs were cultured in Dulbecco's Modified Eagle's Medium (DMEM. Thermo Fisher Scientific, #11965-092), 1 ng/ml human LIF (Cell Signaling, #8911), 100 μM β-mercaptoethanol, 2 mM l-glutamine (Thermo Fisher Scientific, #25030164), 1 × MEM nonessential amino acids (Thermo Fisher Scientific, #11140050). A375, C2C12^*WT*^, C2C12^*Myod1−/−*^, U-118 MG, HCT 116, HepG2 and mouse embryonic fibroblasts (MEFs) from E13.5 embryos were cultured in DMEM. NE-4C cells were cultured in MEM (Gibco, #11090073) containing 1% glutamax (Gibco, #35050061) and 1% MEM non-essential amino acids. NCI-H460 was culture in PRMI-1640 (Thermo Fisher Scientific, #11875093), U-2OS in McCoy’s 5A (Thermo Fisher Scientific, #16600082), and SW480 was cultured in Leibovitz’s L-15 medium (Thermo Fisher Scientific, #41300039). Mesenchymal stem cells (MSCs) from rat bone marrow was cultured in MSC complete medium (Cellbank of Chinese Academy of Sciences. Cat. No.: #SCSP-615). All culture media except MSCs were supplemented with 10% fetal bovine serum (FBS. Gibco, #10099141) (15% FBS for mESCs) and with 50 U/ml penicillin and 50 μg/ml streptomycin. Cells were cultured at 37 °C with 5% CO_2_. mESCs were cultured in petri dishes coated with 0.1% gelatin, and NE-4C was cultured in dishes coated with 10 μg/ml poly-d-lysine (PDL. Sigma-Aldrich, #P0899). Besides culturing cells in regular media, cells were also cultured in a serum-free medium, which is described below.

A375 (Cat. No.: #SCSP-533), HCT 116 (Cat. No.: #TCHu 99), U-118 MG (Cat. No.: #TCHu216), SW480 (Cat. No.: #TChu172), NCI-H460 (Cat. No.: #TCHu205), U-2OS (Cat. No.: #TCHu 88), HepG2 (Cat. No.: SCSP-510), NE-4C (Cat. No.: #SCSP-1501) and MSCs (Cat. No.: #SCSP-402) were purchased from the Cellbank of Chinese Academy of Sciences (Shanghai, China). C2C12 was a gift from Dr. Minsheng Zhu, and mESCs were a gift from Dr. Jinzhong Qin at the Model Animal Research Center of Nanjing University. All cells were detected free of mycoplasma contamination with PCR. Cancer cell lines were authenticated with short tandem repeat profiling, which was performed in September 2019 and May 2020, respectively. Cells with fewer than eight passages were used for experiments.

### In vitro differentiation of mESCs, NE-4C and C2C12^*Myod1*−/−^ cells and neurosphere formation of various cell types

Primitive NSCs (primNSCs) were differentiated from mESCs by culturing mESCs in defined serum-free medium Ndiff 227 (CellArtis, #Y40002) at 37 °C with 5% CO_2_, which was used for differentiation of ESCs into neuroectodermal cells [28]. primNSCs began to form free-floating neurospheres in medium 4 days later and the neurospheres grew larger with continuing culture. MEFs, C2C12^*WT*^, C2C12^*Myod1−/−*^, NE-4C, A375, HCT 116, U-118 MG, NCI-H460, SW480, U-2OS and MSCs were also cultured in this medium to test their ability in neurosphere formation. All cells were plated at a density of 1 × 10^5^/cm^2^. Except SW480, U-2OS, MSCs, MEFs and C2C12^*WT*^ that could not form a spherical structure in the medium, other cell types began to form a spherical structure after usually 4 days of culture. The spherical structures gradually grew larger and started to float freely (except U-118 MG, which remained attached to bottom) with the extension of culture time. For injection of mESC-derived primNSCs into immunodeficient nude mice, neurospheres were dissociated by trituration.

To determine cell tumorigenicity after differentiation, NE-4C and C2C12^*Myod1*−/−^ cells were treated with retinoic acid (RA) at 1 μM for 72 h, while cells treated with DMSO were used as a control. After treatment, cells were used for assays of immunoblotting, soft agar colony formation, and subcutaneous injection into nude mice.

### Isolation of cortical cells and fibroblasts from mouse E13.5 embryos

All experiments with animals (including xenograft tumor assays below) in the study (Animal protocol No.: CY04) were approved by and in accordance with the guidelines of the Institutional Animal Care and Use Committee (IACUC) at the Model Animal Research Center of Nanjing University. For isolation of embryonic cortical cells, C57BL/6 mice with gestation on day 13.5 were sacrificed and embryos were resected. Brains were excised from embryos with forceps, and then cortical tissues were separated after removal of meninges and the ganglionic eminences. Cortical tissues were washed with ice-cold DMEM medium thrice. Afterwards the tissues were placed in 1 ml of 0.25% trypsin–EDTA and incubated in a water bath at 37 °C for 10 min. Trypsinization was terminated by addition of three volumes of DMEM containing 10% FBS. Cortical cells were then collected by centrifugation at 1000 rpm for 5 min at room temperature, washed twice with PBS, and re-suspended in PBS. Cortical cells were cultured in serum-free medium for neurosphere formation and immunofluorescence. Part of the cells were also homogenized in TRIzol (ThermoFischer Scientific, #15596018) and stored at − 80 °C for later use for total RNA extraction.

For isolation of MEFs, the head regions, limbs and visceral organs were removed from E13.5 embryos. The remaining trunk regions were minced into fine pieces, which were suspended in 1 ml of 0.25% trypsin–EDTA, and incubated in a 37 °C water bath for 15 min. Afterwards, three volumes of DMEM containing 10% FBS were used to stop trypsin activity. Cells were collected by centrifugation, resuspended and plated into petri dishes for culture in complete MEF medium. MEFs were used for injection into nude mice, or used for culture in serum-free medium as a control for neurosphere formation assay.

### Xenograft tumor assay

Immunodeficiency nude Foxn1^nu^ mice with an age of five to six weeks were purchased from the National Resource Center for Mutant Mice (Nanjing, China) and maintained in a specific-pathogen-free facility. Different numbers of cells, depending on cell types as listed in Additional file 1: Tables S1 and S2, were suspended in 100 μl of sterile PBS, and injected subcutaneously into the dorsal flank of mouse. Tumor growth was measured periodically. The period for tumor growth varied depending on cell types, as listed in Additional file 1: Tables S1 and S2. Afterwards, mice were sacrificed, and tumor tissues were excised and attached host tissues were carefully removed, and then tumor tissues were weighed. Significance of difference in tumor weight between two groups of mice were calculated with two-tailed Student’s *t*-test. Tumor volume was calculated using the formula: length × width^2^/2. The significance of difference in tumor volumes between two groups was calculated using two-way ANOVA followed by Bonferroni/Dunn (ANOVA-Bonferroni/Dunn) tests.

After measurement, tumor tissues were cut into two parts. One part was homogenized in TRIzol for total RNA extraction; the other part was fixed with 4% paraformaldehyde and embedded in paraffin, and used for histological examination with hematoxylin and eosin (HE) staining and immunohistochemistry analysis. Sections were viewed and photographed with a microscope (Olympus BX53).

NE-4C cells were also injected via tail vein of nude mice to evaluate their tumorigenicity in different regions of animal body. Each 1 × 10^6^ cells were suspended in 100 μl of sterile PBS and injected into mice via tail vein. Mice were sacrificed in one to three months after injection, depending on the progression of tumor growth.

### Immmunoblotting (IB)

Whole cell lysates (WCL) were prepared as described [11]. IB detection of protein expression was performed with WCL using conventional SDS-PAGE, and signals were revealed with a Western blotting substrate (Tanon, #180-501). Primary antibodies were (Fold of antibody dilution indicated): β-Act (Cell Signaling, #4970. 1:10,000), Fgfr1 (Cell Signaling, #9740. 1:1000), Hes1 (Cell Signaling, #11988. 1:1,000), Map2 (Cell Signaling, #8707. 1:1,000), Msi1 (Cell Signaling, #5663. 1:1,000), Myc (Cell Signaling, #13987. 1:2,000), Myod1 (Novus Biologicals, #NB100-56511. 1:1,000), Pcna (Cell Signaling, #13110. 1:3000), Sox1 (Abcam, #ab109290. 1:1,000), Sox2 (Cell Signaling, #23064. 1:1,000), Sox9 (Cell Signaling, #82630. 1:2000), Syn1 (Cell Signaling, #5297. 1:1000).

### Immunohistochemistry (IHC)

Proteins marking different tissue differentiation in tumors were detected with immunohistochemistry using conventional method. Briefly, sections were deparaffinized and rehydrated with three washes of xylene for 5 min each, two washes of 100% ethanol for 10 min each, two washes of 95% ethanol for 10 min each, and two washes in dH_2_O for 5 min each. Antigen unmasking was performed by steaming slides in 0.01 M sodium citrate buffer (pH 6.0) for 25 min. Afterwards, sections were washed in dH_2_O for three times, followed by incubation in 3% H_2_O_2_, and washed again in dH_2_O. After blocking, sections were incubated with primary antibodies diluted in antibody diluent overnight at 4 °C. Primary antibodies were removed and sections were washed, and then incubated with biotinylated secondary antibody (1:500). DAB substrate was used for signal visualization. Cell nuclei were counter-stained with hematoxylin. Primary antibodies were: ACP5 (Novus Biologicals, #NBP2-45293. 1:250), ACTA2 (Abclonal, #A11111. 1:250), AFP (Cell Signaling, #4448. 1:250), BGLAP (Abclonal, #A6205. 1:500), CTSK (ABclonal, #A5871. 1:500), KRT5 (Cell Signaling, #71536. 1:200), MAP2 (Abcam, #ab183830. 1:1200), PAX6 (Abcam, #ab195045. 1:200), SOX1 (Abcam, #ab109290. 1:200), SOX9 (Cell Signaling, #82630. 1:200), and anti-human nuclei antibody (Sigma-Aldrich, #MAB1281. 1:50). Negative controls were performed in parallel in which primary antibodies were not used.

### Immunofluorescence (IF)

IF detection of marker proteins in cells, myotubes, neurospheres or neurosphere-like structures derived from cancer cells was performed exactly as described [11]. Primary antibodies were Myc (Cell Signaling, #13987. 1:400), Msi1 (Cell Signaling, #5663. 1:200), Sox1 (Abcam, #ab109290. 1:250), Myod1 (Novus Biologicals, #NB100-56511; Cell Signaling, #13812. 1:200), Oct4 (Cell Signaling, #83932. 1:500), Pax3 (Abcam, #ab15717. 1:500), Myoglobin (Cell Signaling, #25919. 1:200), Myosin heavy chain (R&D systems, #MAB4470. 1:200), Map2 (Cell Signaling, #8707. 1:200), Synapsin-1 (Cell Signaling, #5297. 1:200). Secondary antibodies were anti-mouse IgG (FITC-conjugated) (Sigma-Aldrich, #F9137. 1:1,000), Alexa Fluor R 568 donkey anti-rabbit IgG (H+L) (Invitrogen, #A10042. 1:1,000), and Cy3-AffiniPure donkey anti-goat IgG (H+L) (Jackson ImmunoResearch Labs, #705-165-147. 1:1,000), anti-mouse or rabbit Alexa Flour 594 (ThermoFisher Scientific, #A21207, #A21203. 1:500), anti-mouse or rabbit Alexa Fluor 647 (ThermoFisher Scientific, #A31573, #A31571. 1:500). Cell nuclei were counterstained with DAPI. Afterwards slides were rinsed, and coverslips were mounted with anti-fade mounting medium (Invitrogen, #S36936). Cells were viewed with a fluorescence microscope (Zeiss LSM 880).

### Total RNA preparation and reverse transcriptase-quantitative polymerase chain reaction (RT-qPCR)

Cells or tumor tissues were homogenized in TRIzol. Total RNAs were extracted following the protocol provided by the manufacturer. cDNAs were synthesized from the total RNAs using the HiScript II 1st Strand cDNA Synthesis Kit (+gDNA wiper) (Vazyme, #R212-01/02), which contains reagent for removal of genomic DNA contamination. qPCR was performed on a LightCycler® 96 system (Roche). Amplification parameters were as follows: one cycle of pre-denaturation at 95 °C for 5 min, followed by 40 cycles of denaturation at 95 °C for 10 s, annealing and extension at 60 °C for 30 s, and an additional cycle for melting curve. Transcription level of β*-Act/*β*-ACT* was detected as a loading control. Significance in changes of gene expression was calculated based on experiments in triplicate using two-tailed Student’s *t*-test. Final results were presented as histograms with relative units of transcription levels. Primers for RT-qPCR were listed in Additional file 1: Table S3.

### Gene knockout in C2C12 cells using CRISPR/Cas9

gRNAs were designed from the first exon of mouse *Myod1* gene using an online design tool (crispr.mit.edu). gRNA target oligos were cloned to the *Bsm*BI site of lentiCRISPRv2 vector. Lentivirus packaging was performed as described [11]. C2C12 cells were infected with lentiviral particles containing the gRNA target and selected with puromycin (1 μg/ml) for 3 days. The remaining cells were plated in 10-cm culture dishes in a density that allowed single cell picking. Single cells were grown in 48-well plates, and then transferred to 24-well plates when cells grew to a high confluency. Afterwards DNA was extracted, and *Myod1* gene was PCR-amplified and assayed with agarose gel electrophoresis. Clones with an extra smaller DNA band were considered as potentially mutated clones and their amplified *Myod1* gene was further sequenced. Amplified PCR products with two bands also showed two heterozygous peaks in sequencing electropherograms, meaning possible deletion in one allele of *Myod1* gene. Cell clones with heterozygous deletions were passaged, picked again for single cells. These single cells were cultured, DNA extracted and gene sequenced again, until a cell clone with homozygous gene deletion was identified. Two pairs of primers were used for genotyping: F0: ACTCCTCTGACAGGACAGGA, R0: TCTCGAAGGCCTCATTCACT; F1: TACTGTTGGGGTTCCGGAGT, R1: GCACATGCTCATCCTCACGA.

### Genomic DNA preparation

Cells were collected by trypsinization and washed in PBS for twice. Each 300 μl of lysis buffer (10 mM Tris–HCl (pH 8.0), 0.4 M NaCl, 35 mM SDS, 2 mM EDTA) was added to re-suspend cells, followed by addition of 5 μl of proteinase K at 20 μg/ml. The mixture was incubated in waterbath at 55 °C for 4 h. Flocculent genomic DNA was precipitated by addition of 700 μl of absolute ethanol.

### Transcriptomic assay with RNA sequencing (RNA-seq)

Transcriptomic profiles of wild type and *Myod1* knockout C2C12 cells were analyzed with RNA-seq. Total RNAs of C2C12^*WT*^ and C2C12^*Myod1*−/−^ cells, each in triplicate, were prepared with TRIzol. Briefly, total RNAs were quantified and rRNAs were removed. After fragmentation of the remaining total RNAs, sequencing libraries were constructed with NEBNext® Ultra™ RNA Library Prep Kit for Illumina® (New England Biolabs, #E7530S), and sequenced with an Illumina NovaSeq platform. Raw data were processed for quality control and cleaned up to generate clean data, which were mapped against the mouse reference genome GRCm38 with Hisat2/Tophat software. Transcript sequence reads were assembled and quantified using Stringtie software. Differentially expressed genes (DEGs) were calculated with limma package. A gene was considered as differentially expressed if |log_2_FC|≥ 1 and meanwhile p-Value ≤ 0.05. Enrichment analyses were made using software KOBAS (http://kobas.cbi.pku.edu.cn), which covers seven pathway databases (KEGG Pathway, PID Curated, PID BioCarta, PID Reactome, BioCyc, Reactome, and Panther), five human disease databases (OMIM, KEGG Disease, FunDO, GAD, and NHGRI), and the Gene Ontology database. Sequencing, signal processing and data analyses were performed by Beijing CapitalBio Technology Co., Ltd. (Beijing, China). RNA-seq data were deposited to the Gene Expression Omnibus (GEO) under accession number GSE137507.

### Myotube formation and neuronal differentiation of wild type and gene knockout C2C12 cells

For detecting muscle differentiation potential, C2C12^*WT*^ and C2C12^*Myod1*−/−^ cells were cultured in DMEM containing 2% horse serum (Sangon Biotech, #E510006). For detecting neuron differentiation potential, C2C12^*WT*^ and C2C12^*Myod1*−/−^ cells were first cultured in Ndiff 227 medium for 6 days. After dissociation of C2C12^*Myod1*−/−^ cells, both C2C12^*WT*^ and C2C12^*Myod1*−/−^ cells were treated with 2 μM retinoic acid (RA) for 24 h.

### Electroporation

In order to rescue C2C12^*Myod1*−/−^ phenotype by introducing extraneous cDNA encoding MYOD1 into the cells, human MYOD1 cDNA was PCR-amplified from the vector pDONER223-hMYOD1 (Cat. No.: G152873. Purchased from YouBio, Changsha, China) and subcloned to the sites ClaI/XbaI of pCS2 + eGFPmcs vector using primers Forward: ccgg atcgat CAG GAT ATG GAG CTA CTG TCG C, and Reverse: ccgg tctaga GAG CAC CTG GTA TAT CGG GTT G. The resulting plasmid encodes a fusion protein MYOD1-eGFP. To introduce the plasmids into C2C12^*Myod1*−/−^ cells, 4 μg of either empty vector or MYOD1-eGFP plasmid was mixed with 1 × 10^6^ cells in 200 μl of electroporation buffer in the Cell Line Nucleofector™ Kit V (Lonza. Cat. No.: #VCA-1003). Cuvette containing the mixture was placed in a Nucleofector™ 2b device (Lonza. Cat. No.: #AAB-1001) and electroporation was performed with program B-032. Afterwards, cells were re-suspended in DMEM medium plus 10% FBS. Twenty-four hours after electroporation, cells were cultured in DMEM containing 2% horse serum (Sangon Biotech, #E510006) for 5 days to detect the rescuing effect in C2C12^*Myod1*−/−^ cells. Moreover, C2C12^*Myod1*−/−^ cells that were replenished with MYOD1 were also tested for their tumorigenicity using soft agar colony formation and xenograft assays.

### Gene conservation, tissue expression analysis and association analysis

A series of genes were analyzed for their conservativeness and expression patterns in tissues, in particular embryonic tissues.

We checked embryonic tissue expression of neural related genes (Additional file 2: Table S4 and Additional file 3: Table S5), and the conservativeness of their protein sequences between typical vertebrate species and unicellular and multicellular animals that represent most closely the evolution from unicellularity to multicellularity. Neural related proteins include about 2600 proteins expressed in human neuron, which were retrieved from GO database under the term ‘neuron’ for the species ‘*Homo sapiens*’ (Additional file 4: Table S6), and other proteins whose genes are specifically expressed or at least enriched in neural tissues in zebrafish, *Xenopus* or mouse embryos. Tissue expression of these genes was primarily determined by RNA whole mount in situ hybridization in *Xenopus* or zebrafish embryos, as described in the *Xenopus* Xenbase (http://www.xenbase.org/) and zebrafish ZFIN databases (https://zfin.org). Tissue expression in mouse was mainly searched from mouse database MGI (http://www.informatics.jax.org/). When expression pattern for a gene is not available in these databases, gene expression in mouse tissues was also determined according to description in the database Emage (http://www.emouseatlas.org/emage/home.php). In very rare cases, gene expression patterns in chicken were determined based on description in literatures. Expression only in neural precursor tissues, including neural ectoderm, neural plate, neural folds, neural tube or neural crest during early embryonic development, or later in central nervous system (CNS) or sensory nervous systems (including visual, auditory and olfactory systems) was considered as neural specific. Expression was mainly in the neural tissues above but with weak expression in other tissues, for example, a gene with strong expression in the CNS and weak expression in somites in *Xenopus* tailbud embryos, was considered as enriched neural expression. Embryonic tissue expression of genes for the about 2600 human neuronal proteins (Additional file 4: Table S6) was also searched against these databases to determine whether they are neural specific or not.

To determine how these neural related genes are conserved during animal evolution, we compared the protein sequences of these genes with protein or peptide sequences of *Monosiga brevicollis* (*M*. *brevicollis*), *Amphimedon queenslandica* (*A*. *queenslandica*) and *Trichoplax adhaerens* (*T*. *adhaerens*), whose genomes have been sequenced, using BLAST (Basic Local Alignment Search Tool) sequence similarity searches against the non-redundant (nr) protein databases. The top hits of output sequences of these three lower species with significant similarity (alignment score > 40 for top hits) were vice versa BLASTed against human and mouse protein databases. If the original input of human, mouse, *Xenopus* or zebrafish proteins whose genes with neural expression (either specific or non-specific) matched the BLAST output of human/mouse proteins using *M. brevicollis*, *A. queenslandica* or *T. adhaerens* sequences, then these *M. brevicollis*, *A. queenslandica* or *T. adhaerens* proteins were considered as proteins homologous to neural related proteins in vertebrates.

In order to examine whether the genes in *M. brevicollis* are biased towards certain cell types in vertebrates, we analyzed gene homology between *M. brevicollis* and mammals, and analyzed the expression of conserved genes in vertebrate embryonic tissues. Gene homology was determined by reciprocal sequence comparison between all 9275 protein (peptide) sequences of *M. brevicollis* and human or mouse non-redundant (nr) protein databases using protein BLAST program, as described above. *M. brevicollis* protein (peptide) sequences, their entry numbers and entry names were downloaded from Uniprot (http://www.uniprot.org) (Additional file 5: Table S7). *M. brevicollis* genes were assigned as mammalian homologous genes according to mammalian proteins that share the highest homology (alignment score > 40) with *M. brevicollis* proteins. However, some *M. brevicollis* proteins have highly conserved domains or motifs, for example, those in protein families. It has been difficult to ascertain the homologs based on the conserved motifs or domains. Thus, these proteins were not included as vertebrate homologs. Subsequently, the homologous genes were searched for their expression in mainly embryonic tissues of mouse, *Xenopus* or zebrafish, as described above.

Besides these genes above, we also collected about 7000 genes that are expressed in non-neural tissues or whose expression patterns are unknown (Additional file 6: Table S8). Association between gene conservation, tissue-specific expression and cancer was analyzed with the program KOBAS, as described above.

### Gene length calculation

Transcript names, their accession numbers, numbers of exons and introns, and transcript lengths were retrieved from a dataset for 37559 mRNA-coding human transcripts in the Additional file 2 in Ref. [29]. Altogether 11270 transcripts for 5283 neural genes, 1688 transcripts for 738 genes with both neural and non-neural expression, and 12874 transcripts for 7238 non-neural genes (Additional file 7: Table S9) were retrieved from the dataset. Simple arithmetic average of transcript length was calculated for each of the three gene sets.

### Cell migration/invasion assays

Experiments were performed as described [10]. C2C12^*WT*^ and C2C12^*Myod1*−/−^ cells were subjected to migration/invasion assays without further treatment. Cell migration assay was performed in 24-well transwell plates with inserts of 8-μm pore size (Corning, #3422). 2 × 10^4^ cells suspended in 200 μl of serum-free culture medium were added to the upper compartment, and 500 μl of culture medium containing 10% FBS was added in the lower compartment. Plates were then incubated at 37 °C for a time period, as indicated in the text. Afterwards, cells were fixed with 37% formaldehyde, followed by staining with 0.5% crystal violet for 10 min. Cells that did not migrate were removed. Migrated cells were washed with PBS and photographed.

For cell invasion assay, each 80 μl of Matrigel (Corning, #354234) was diluted in PBS (1:8) and distributed uniformly onto a 24-well transwell insert. 1 × 10^5^ cells were added to Matrigel, which was submerged in the culture medium in the lower compartment. Plates were incubated at 37 °C for time periods as indicated in the text. Later on, cells were treated using the same manner as in the migration assay.

### Soft agar colony formation assay

Colony formation assay was performed as described [10]. The top and bottom layer of agar was 0.35% and 0.7%, respectively, of low melting agarose (BBI, #AB0015). Each 2000 cells were distributed in a well of a 6-well culture plate and cultured for different time periods, as indicated in text. Colonies with a size larger than 25 μm in diameter were counted. Each experiment was performed in triplicate. Significance of difference in colony formation was calculated using two-tailed Student’s *t*-test.

## Results

### ESC-derived primNSCs show tumorigenicity

**Fig. 1:**
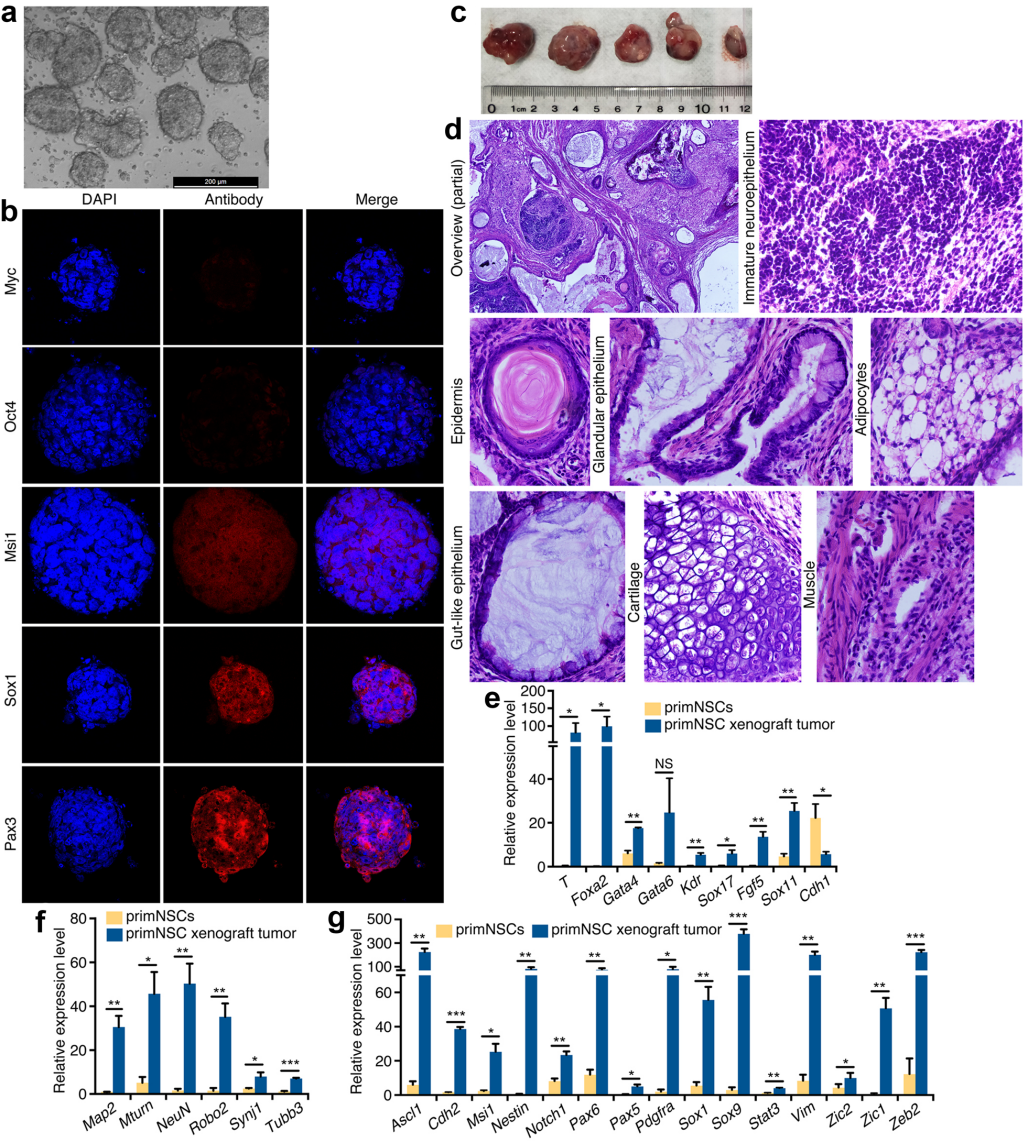
Tumorigenicity and pluripotent differentiation potential of primNSCs. **a** Neural differentiation from mESCs cultured in serum-free medium, as shown by neurosphere formation after 6 days of culture. **b** Characterization of primNSC fate in neurospheres by IF detection of markers for pluripotency and neural stemness. Nuclei were counterstained with DAPI. **c** Tumors formed by injection of 0.5 × 10^6^ of primNSCs. **d** Abundant diversity of tissue types in tumor. Displayed are a partial overview of tissue differentiation in a representative section and a few tissue types derived from ectoderm, endoderm and mesoderm. Original objective magnification: 4× for overview, 40× for specific tissues. **e**–**g** Comparison of expression of tissue or cell type-specific genes between primNSCs and tumor. Gene expression representing non-neural differentiation (**e**), neuronal differentiation (**f**) and neural stemness (**g**) was detected with RT-qPCR. Significance of gene expression change was calculated based on experiments in triplicate using two-tailed Student’s *t*-test. Data are shown as mean ± SEM. *p < 0.05, **p < 0.01, ***p < 0.001. NS: not significant

The ‘neural default state’ of ESCs prompted us to investigate whether ESC-derived NSCs exhibit tumorigenicity. As reported, mouse ESCs (mESCs) differentiated into neuroectodermal cells, the primNSCs [24, 25, 28] after 6 days of culture in serum-free medium, which formed floating neurospheres (Fig. 1a). Immunofluorescence (IF) did not reveal significant expression of ESC markers Myc and Oct4 (Fig. 1b), whereas NSC markers Msi1, Sox1 and Pax3 were detected in neurospheres (Fig. 1b), confirming the gain of NSC fate in mESCs. We tested whether primNSCs had also tumorigenicity. When each 1 × 10^6^ cells were injected, both mESCs and primNSCs formed tumor in all injected nude mice (Additional file 1: Figure S1A and Table S1), but tumor growth rate and weight of mESCs was lower than that of primNSCs (Additional file 1: Figure S1B and C). To confirm whether primNSCs have stronger tumor-initiating capacity, a series of number of cells were injected. Indeed, much fewer primNSCs than ESCs were required for tumor initiation (Additional file 1: Table S2). The results suggest that tumor formation by primNSCs should not be due to the effect of remaining undifferentiated mESCs, and primNSCs are more tumorigenic than mESCs. PrimNSC-derived tumors (Fig. 1c) showed a wide spectrum of phenotypically identifiable tissue types (Fig. 1d), e.g. the neural tissue and keratinized structures derived from ectoderm (Fig. 1d), the endodermal tissues including glandular and gut-like epithelia (Fig. 1d), and mesodermal tissues such as adipocytes, cartilage and muscle (Fig. 1d). Quantitative RT-PCR (RT-qPCR) revealed that genes representing mesodermal (*T*, *Kdr*), endodermal (*Foxa2*, *Gata4/6*, *Sox17*) and ectodermal (*Fgf5*, *Sox11* for primitive ectoderm) differentiation were generally activated in tumor (Fig. 1e). Neuronal differentiation also occurred, as shown by activation of neuronal genes in tumor (Fig. 1f) and the presence of neuropil structure adjacent to immature neuroepithelium (Fig. 1d). Besides, a series of genes representing neural stemness were significantly upregulated in tumor compared with primNSCs (Fig. 1g). Neural stemness genes, which are responsible for self-renewal and differentiation of NSCs/NPCs, play cancer-promoting roles or are upregulated during tumorigenesis [10, 12]. Upregulation of these genes suggests a continuing promotion of tumor growth. This fashion of tissue differentiation in primNSC-derived tumor is reminiscent of ESC-derived teratoma/teratocarcinoma. Hence, primNSCs have tumorigenicity and pluripotent differentiation potential.

### Embryonic NSCs/NPCs also show tumorigenicity and differentiation potential for non-neural tissues

**Fig. 2:**
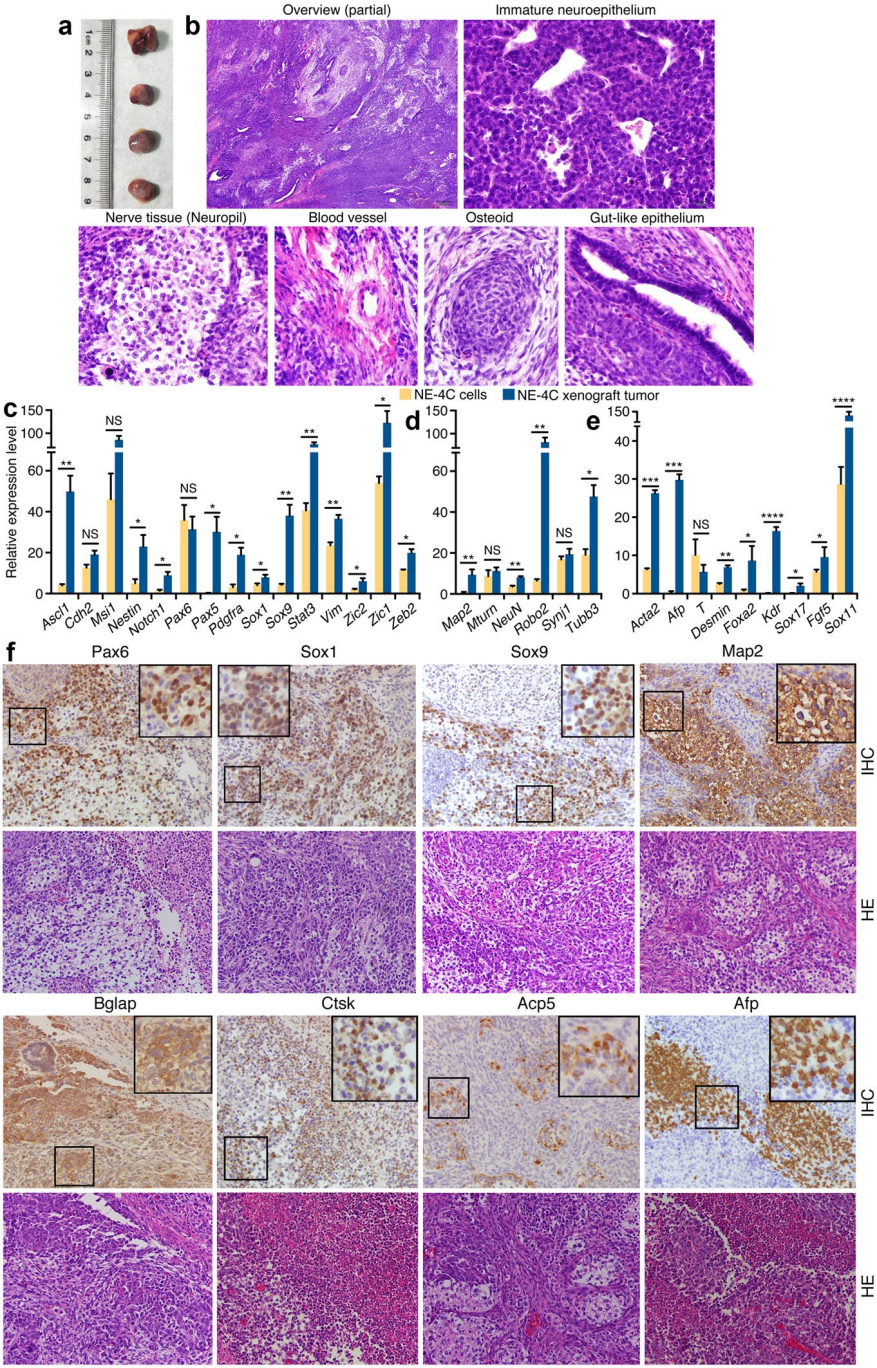
Tumorigenicity and differentiation potential of neural stem cell NE-4C. **a** Tumor formation by NE-4C in nude mice. **b** Tissue differentiation in NE-4C xenograft tumors examined by histology. A partial overview of a section with HE staining and particular tissue types in the section are shown. Original objective magnifications for sections: 4× for overview, 40× for specific tissues. **c**–**e** Differential expression of genes representing neural stemness (**c**), neuronal differentiation (**d**), and mesendodermal tissue differentiation (**e**) in NE-4C cells and tumor, as detected with RT-qPCR. Significance of expression change was calculated based on experiments in triplicate using two-tailed Student’s *t*-test. Data are shown as mean ± SEM. *p < 0.05, **p < 0.01, ***p < 0.001, ****p < 0.0001. NS: not significant. **f** IHC detection of cell/tissue markers in tumor sections. Below the marker panels are corresponding sections stained with HE. Objective magnification: 20×; insets: 40×

We tested whether NSCs/NPCs at later stages of embryonic neural development are still tumorigenic and display differentiation potential. NE-4C is a NSC cell line derived from cerebral vesicles of mouse E9 embryos. The cells injected subcutaneously also formed tumors in nude mice (Fig. 2a; Additional file 1: Table S1). Moreover, injection of the cells via tail vein caused tumor formation in different areas of animal body, e.g. abdominal cavity, legs, etc. (Additional file 1: Figure S2A and B), suggesting that the cells have both tumorigenic and metastatic potentials, and the tumorigenicity is not restricted to a specific environment. Histological examination showed that the tumors contained large areas of blue-staining, immature neuroepithelium-like cells, and a variety of other tissue types (Fig. 2b). There were differentiated nerve tissue (neuropil), mesodermal derivatives such as blood vessels, osteoid tissue, as well as endodermal derivatives including gut-like epithelium (Fig. 2b). Compared with NE-4C cells, the tumor showed a general tendency of upregulation of a series of genes representing neural stemness (Fig. 2c), genes representing neuronal differentiation (Fig. 2d), and genes representing mesodermal (*Acta2*, *T*, *Desmin*, *Kdr*) and endodermal tissue (*Afp*, *Foxa2*) differentiation (Fig. 2e). Immunohistochemistry (IHC) demonstrated strong expression of neural stemness markers Pax6, Sox1 and Sox9 in cells characteristic of undifferentiated neuroepithelial cells, and the neuronal marker Map2 in areas with obvious features of differentiated neural tissue (Fig. 2f). The tumor also expressed the osteoblast marker Bglap and the osteoclast markers Acp5 and Ctsk (Fig. 2f). Ctsk is a marker for macrophage differentiation, too. The endodermal marker Afp was detected significantly (Fig. 2f). Although tissue types of all germ layers could be observed, tissue differentiation and diversity appeared not as abundant as in tumors generated from primNSCs. These results demonstrate that NSCs derived from early brain vesicle have differentiation potential for non-neural lineages. Besides the general roles of neural stemness genes in promoting cancer, expression of Acta2, Desmin or Kdr is frequently observed during cancer progression, and Afp is used as a marker for cancers of the liver, testicles and ovaries. Therefore, this fashion of tissue differentiation and gene expression change is similar to what occurs in cancers.

**Fig. 3:**
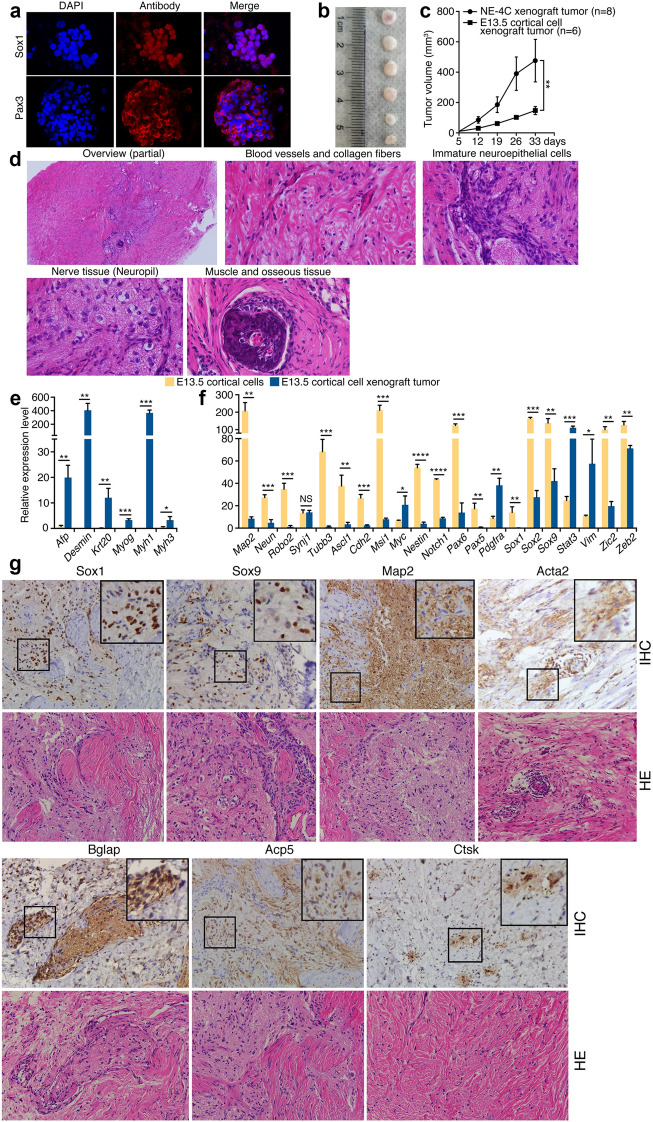
Tumor formation and tissue differentiation by cortical NPCs from E13.5 mouse embryos. **a** IF staining of neural stemness marker expression in neurospheres formed by cortical NPCs cultured in serum-free medium. Cell nuclei were counter-stained with DAPI. **b** Xenograft tumors formed by NPCs in nude mice. **c** Comparison of tumor growth by NE-4C and cortical cells within a same period. Significance of difference in tumor volume between two groups was calculated using two-way ANOVA-Bonferroni/Dunn test. Data are shown as mean ± SEM. *p < 0.05, **p < 0.01, ***p < 0.001, ****p < 0.0001. NS: not significant. **d** An overview and specific types of tissue differentiation in an HE-stained tumor section. Original objective magnifications: 4× for overview, 40× for specific tissues. **e**, **f** Differential expression of genes representing mesendodermal tissue differentiation (**e**), neural stemness and neuronal differentiation (**f**) in NPCs and xenograft tumors, as detected with RT-qPCR. Significance of expression change was calculated based on experiments in triplicate using two-tailed Student’s *t*-test. Data are shown as mean ± SEM. *p < 0.05, **p < 0.01, ***p < 0.001, ****p < 0.0001. NS: not significant. **g** IHC detection of cell/tissue markers in tumor sections. Below the marker panels are corresponding sections stained with HE. Objective magnification: 20×; insets: 40×

Next we examined the NPCs from mouse embryonic cortices at E13.5, at which stage the NPCs are more differentiated than those at E9. Disaggregated cortical cells formed neurospheres in serum-free medium, which expressed neural stemness markers Sox1 and Pax3 (Fig. 3a). These cells formed tumors in nude mice (Fig. 3b; Additional file 1: Table S1). Tumor growth of cortical NPCs was much slower than that of NE-4C cells (Fig. 3c), even the injected cell number of the former cells was more than the latter (Additional file 1: Table S1). The tumor was dominantly composed of eosinophilic tissues, in particular tissues that contain large bundles of collagen fibers (Fig. 3d). In this background, other tissues could be identified, including blood vessels, undifferentiated neuroepithelial cells, and differentiated nerve tissue (Fig. 3d). A piece of osseous tissue, which was adjacent to muscle tissue, was present in tumor (Fig. 3d). The tumor displayed a strong activation of genes for mesodermal (*Desmin*, *Myog*, *Myh1* and *Myh3*) and endodermal (*Afp*, *Krt20*) tissue differentiation (Fig. 3e). Transcription upregulation was also observed for neural stemness genes, *Pdgfra*, *Stat3* and *Vim* (Fig. 3f). Nevertheless, genes for neuronal differentiation (*Map2*, *Neun*, *Robo2*, *Tubb3*) and more genes for neural stemness (*Ascl1*, *Cdh2*, *Msi1*, *Nestin*, *Notch1*, *Pax5/6*, *Sox1*, *Sox2*, *Sox9*, *Zic2*, *Zeb2*) were reduced (Fig. 3f). In tumor sections, expression of Sox1, Sox9, and Map2 was obvious in undifferentiated cells and differentiated neural tissue (Fig. 3g). The marker for smooth muscle Acta2 and markers for osteoblasts and osteoclasts Bglap, Acp5 and Ctsk, were detected in restricted areas or scattered cells (Fig. 3g). These data show that NPCs, which are undergoing neuronal differentiation at E13.5 during normal neural development, differentiate into different tissues in a non-native environment. The general tendency of decreased expression in neural stemness genes was correlated the weak capability of tumor growth. Thus, like primNSCs and NE-4C cells, NPCs from developing brains also exhibit differentiation potential for non-neural tissues.

As a control, MEFs isolated from E13.5 embryos did not form tumors in nude mice, even in an extended period after injection (Additional file 1: Table S1). Tumorigenicity was not observed for MSCs after 114 days post injection (Additional file 1: Figure S3 and Table S1). Myoblasts did not cause tumor formation, either (see text below). Therefore, tumorigenicity and differentiation potential are properties of NSCs/NPCs.

### Cancer cells display the ability of neurosphere formation and differentiation potential, similar to NSCs/NPCs

**Fig. 4:**
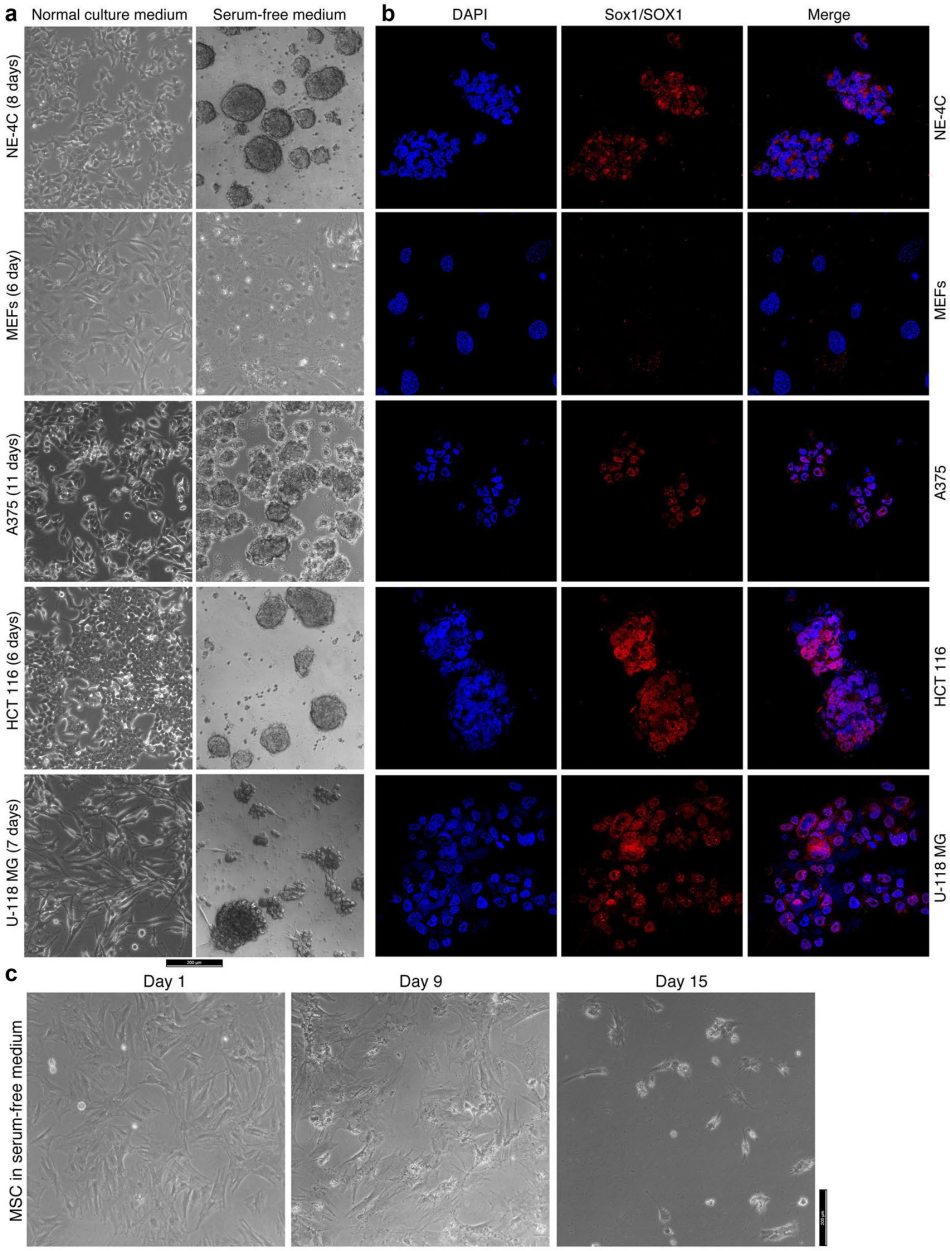
Difference in ability of formation of neurosphere-like structures by different cells in serum-free culture. **a** Different phenotypes of cancer cell lines when cultured in their normal culture medium and in serum-free medium. NE-4C neural stem cells and MEFs were cultured as controls. **b** IF detection of the neural stemness marker Sox1/SOX1 in these cells that were cultured in serum-free medium. Nuclei were counterstained with DAPI. **c** Culture of MSCs in serum-free medium

Free-floating neurosphere formation in NSC serum-free medium is a feature of NSCs, as shown by primNSCs (Fig. 1a). NE-4C cells grew in a monolayer when cultured in its regular medium. The cells also formed neurospheres in serum-free medium (Fig. 4a). However, MEFs did not form any spherical structures, either in their regular medium or in serum-free medium (Fig. 4a). Cancer cells, such as melanoma cell line A375, colorectal carcinoma cell line HCT 116 or glioblastoma cell line U-118 MG, grew in monolayers in their respective regular media. In serum-free medium, nevertheless, A375 and HCT 116 formed free-floating neurosphere-like structures (Fig. 4a). U-118 MG also formed a spherical structure, but not free-floating (Fig. 4a). IF showed the expression of NSC marker Sox1 in NE-4C neurospheres but not in MEFs (Fig. 4b). This marker was detected in the spherical structures formed by cancer cells (Fig. 4b), supporting the similarity between cancer cells and NSCs/NPCs. Many types of tissue stem/progenitor cells form spheres in serum-free media. The media are usually cell-specific and not exchangeable. For example, rat bone marrow mesenchymal stem cells (MSCs) form spheres in specific medium, but could not in serum-free medium used in current study (Fig. 4c). Therefore, sphere formation in NSC serum-free medium indicates neural stemness.

**Fig. 5:**
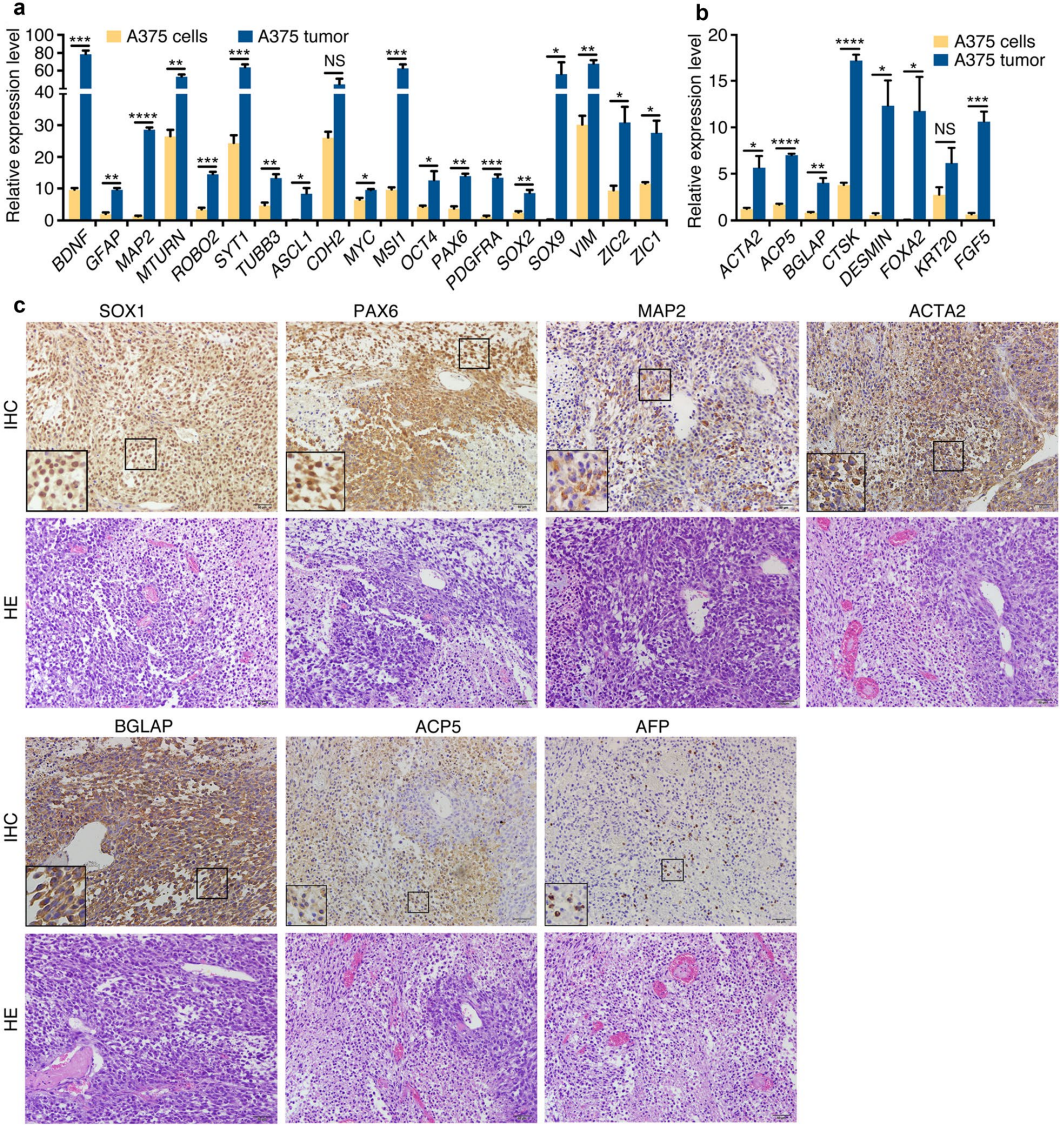
Tissue differentiation in A375 xenograft tumors. (A and B) RT-qPCR detection of expression of genes for neuronal and neural stemness (**a**) and genes for mesodermal or/and endodermal tissue differentiation (**b**) in cells and tumors. Significance of gene expression change was calculated based on experiments in triplicate using two-tailed Student’s *t*-test. Data are shown as mean ± SEM. *p < 0.05, **p < 0.01, ***p < 0.001, ****p < 0.0001. NS: not significant. **c** IHC detection of cell/tissue markers in tumor sections. Below the marker panels are corresponding sections stained with HE. Objective magnification for sections: 20×; insets: 40×

We investigated whether these cancer cells could show differentiation potential. These cells formed tumors in nude mice (Additional file 1: Table S1). Compared with A375 cells, A375 xenograft tumor displayed an upregulation for glial and neuronal genes (*GFAP*, *BDNF*, *MAP2*, *MTURN*, *ROBO2*, *SYT1* and *TUBB3*) and neural stemness genes (*ASCL1*, *MSI1*, *PAX6*, *PDGFRA*, *SOX9*, *VIM*, *ZIC1/2*) (Fig. 5a). Pluripotency genes *MYC*, *OCT4* and *SOX2* were also upregulated (Fig. 5a). Actually, these genes are neural specific during embryonic development in *Xenopus* and mouse [30] besides their earlier expression in blastocysts. Moreover, genes representing mesodermal or/and endodermal tissue differentiation (*ACP5*, *ACTA2*, *BGLAP*, *CTSK*, *DESMIN*, *FOXA2*) were activated in tumor (Fig. 5b). IHC also identified strong expression of neural stemness markers SOX1 and PAX6 in cells rich in nuclei, similar to immature neuroepithelial cells (Fig. 5c). Neuronal marker MAP2 was detected among neuroepithelial-like cells (Fig. 5c). Intense staining was also observed for ACTA2, BGLAP and ACP5 (Fig. 5c). The endodermal marker AFP was detected in scattered cells (Fig. 5c).

**Fig. 6:**
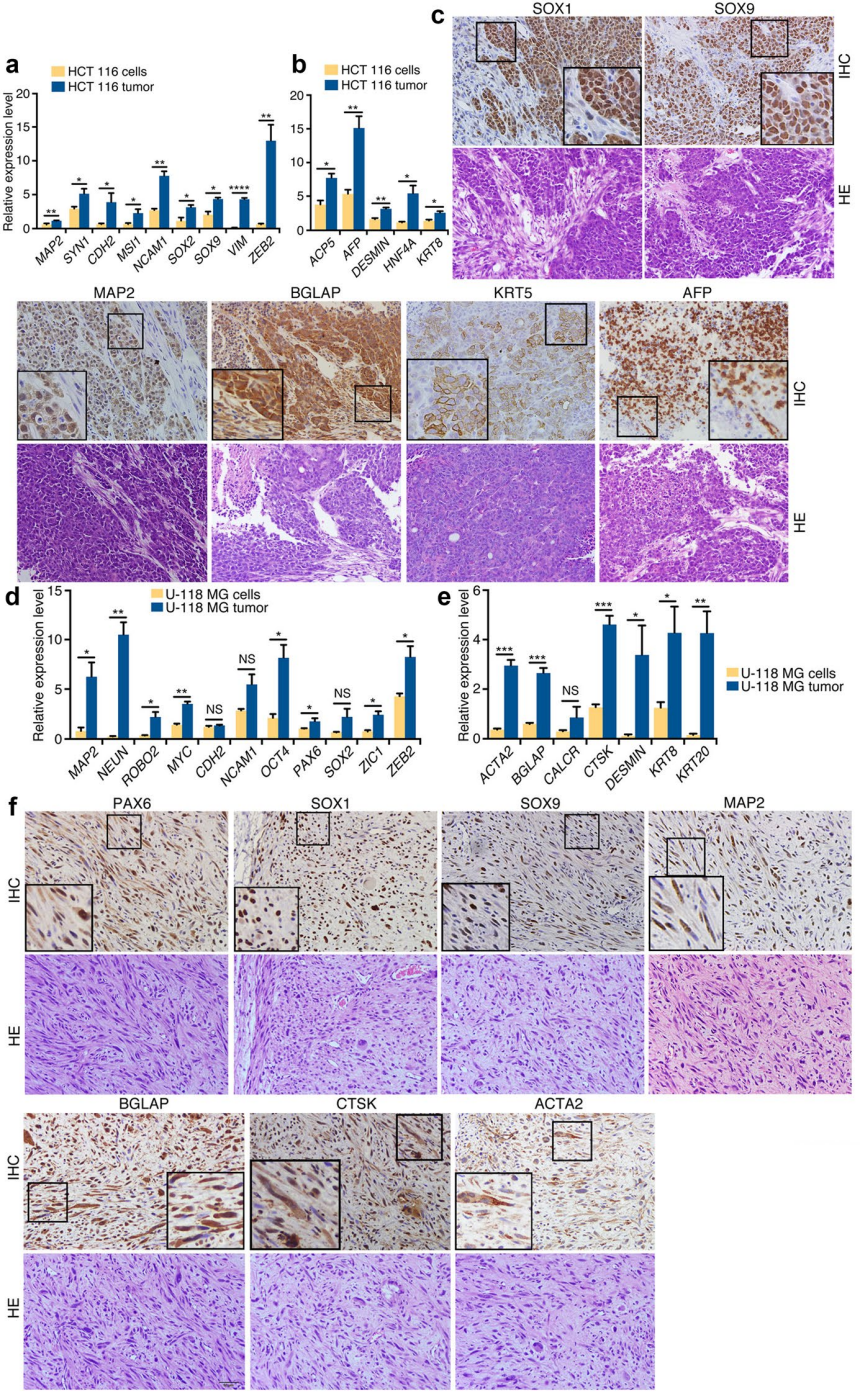
Tissue differentiation in HCT 116 and U-118 MG xenograft tumors. **a**, **b** RT-qPCR detection of expression of genes for neuronal and neural stemness (**a**) and genes for mesodermal or/and endodermal tissue differentiation (**b**) in HCT 116 cells and tumors. **c** IHC detection of cell/tissue markers in HCT 116 tumor sections. Below the panels for marker expression are corresponding sections stained with HE. **d**, **e** RT-qPCR detection of expression of genes for neuronal and neural stemness (**d**) and genes for mesodermal or/and endodermal tissue differentiation (**e**) in U-118 MG cells and tumors. **f** IHC detection of cell/tissue markers in U-118 MG tumor sections. Below the panels for marker expression are corresponding sections stained with HE. In (**a**, **b**) and (**d**, **e**), significance of gene expression change was calculated based on experiments in triplicate using two-tailed Student’s *t*-test. Data are shown as mean ± SEM. *p < 0.05, **p < 0.01, ***p < 0.001, ****p < 0.0001. NS: not significant. In **c** and **f**, objective magnification for sections: 20×; insets: 40 ×

Xenograft tumor of HCT 116 showed an increased expression of neuronal genes *MAP2* and *SYN1*, neural stemness genes *CDH2*, *MSI1*, *NCAM1*, *SOX2/9*, *VIM* and *ZEB2* (Fig. 6a), and genes for mesodermal and endodermal tissues (*ACP5*, *AFP*, *DESMIN*, *HNF4A* and *KRT8*) (Fig. 6b). IHC showed more detailed information on tissue types in the tumor. Large areas of intense staining of SOX1, SOX9 and MAP2 (Fig. 6c) suggested that cells with neural stemness and neuronal features were among the major cell types in tumor. Strong expression of BGLAP was present. Besides, KRT5 and AFP expression was seen in many cells (Fig. 6c). Gene expression comparison between U-118 MG cells and the tumor showed an increased expression of neuronal and neural stemness genes *MAP2*, *NEUN*, *ROBO2*, *PAX6*, *ZIC1*, *ZEB2*, as well as *MYC* and *OCT4* in tumor (Fig. 6d). Likewise, Gene expression for mesodermal and endodermal tissues *ACTA2*, *BGLAP*, *CTSK*, *DESMIN* and *KRT8/20* was enhanced (Fig. 6e). In agreement, expression of PAX6, SOX1, SOX9, MAP2, BGLAP, CTSK, ACTA2, which represent undifferentiated and differentiated neural cells, and different mesodermal cell types, was observed with IHC (Fig. 6f). We also stained the tumor sections with an antibody specific for human cells. Staining signal was universal in sections of tumors of cancer cells (Additional file 1: Figure S4A), but not present in the NE-4C xenograft tumor section (Additional file 1: Figure S4B). Considering together that transcription was detected specifically for human genes, different cell or tissue types detected in tumors were derived from injected cancer cells, but not from host mouse tissues. Hence, these cancer cells display the property of NSCs/NPCs in their capacity of neurosphere formation and differentiation potential.

**Fig. 7:**
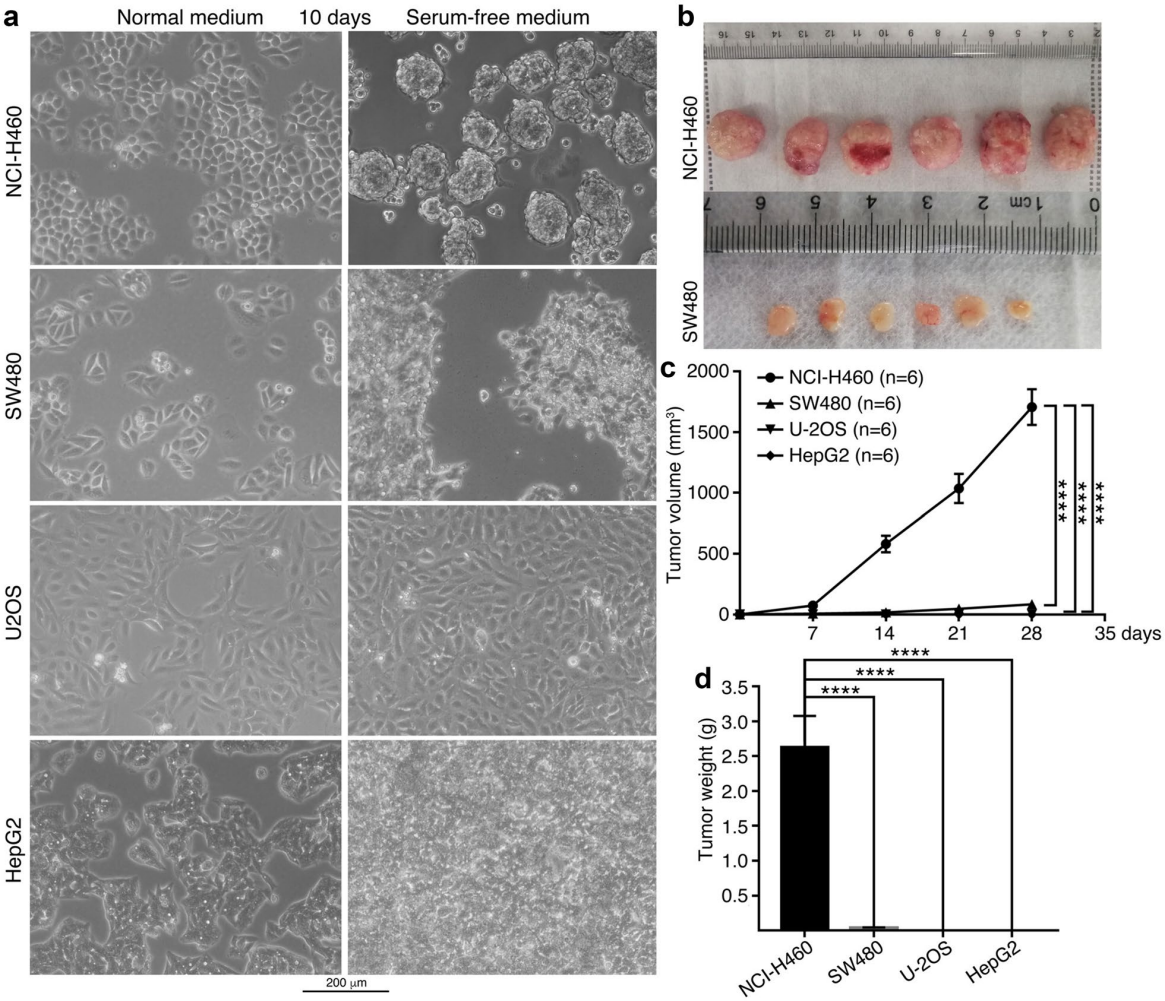
Correlation between capability of neurosphere formation and tumor initiation of cells. **a** Culture of different cancer cells in serum-free medium for neurosphere formation. Cells cultured in their respective normal media served as a control. **b** Tumors formed in nude mice by injected cells. **c**, **d** Comparison of growth rate (**c**) and weight (**d**) of tumors derived from different cells. Significance of difference in tumor volume between two groups of mice was calculated using two-way ANOVA-Bonferroni/Dunn test, and significance of difference in tumor weight was calculated using two-tailed Student’s *t*-test. Data are shown as mean ± SEM. ****p < 0.0001

Different cancer cells showed a difference in their ability of neurosphere formation. Among lung cancer cell NCI-H460, colorectal cancer cell SW480, osteosarcoma cell U-2OS and hepatocellular carcinoma cell HepG2, NCI-H460 formed neurospheres, but other cells didn’t in serum-free medium (Fig. 7a). SW480 showed cell morphological change. U-2OS and HepG2 did not even display change in cell morphology, with only an increase in cell density (Fig. 7a). This difference in neurosphere formation indicates different degrees of neural stemness in these cells. When the same numbers of cells were injected into nude mice, NCI-H460 and SW480 formed tumors (Fig. 7b; Additional file 1: Table S1). Nevertheless, tumors of NCI-H460 grew faster and larger than those of SW480. U-2OS and HepG2 did not form tumors in this assay (Fig. 7b–d). The results might suggest that the degree of neural stemness in cancer cells is proportional to their capacity of tumor initiation. Cancer cells without tumor formation could mean that they are not sufficient for tumor initiation but still possess malignant features like fast proliferation, clonogenicity, etc., or possibly exhibit tumorigenicity under a more sensitive condition, such as in more severely immunodeficient mice.

### *Myod1* knockout in myoblast cells leads to a neural phenotype and confers tumorigenicity in cells

Now that NSCs/NPCs display tumorigenicity and can act as an initial state for non-neural tissue differentiation, we explored whether the process could be reversed in somatic cells. C2C12 is a mouse myoblast cell line used for investigating muscle differentiation. We employed CRISPR/Cas9 to knock out *Myod1*, which encodes a transcription factor critical for myogenesis, in C2C12 cells. After lentiviral transfection of the vector containing the sgRNA, which corresponds 338–357 bp in the first exon of *Myod1* gene (Additional file 1: Figure S5A), cells were selected with puromycin. After serial passaging and genotyping, we identified a clone showing homozygous deletion for *Myod1* gene. PCR with a pair of primers F0/R0 generated a correct product (550 bp) in wild type gene (Additional file 1: Figure S5A and B), but generated a shorter product in the selected clone (Additional file 1: Figure S5B). A pair of nested primers that should generate a product of 323 bp in wild-type cells (Additional file 1: Figure S5A) produced no product from the genomic DNA of the selected clone (Additional file 1: Figure S5C). Sequencing showed a 209 bp deletion in *Myod1* gene, ranging from 134 to 342 bp downstream the start codon, with the sgRNA target sequence locating at the beginning of the missing sequence (Additional file 1: Figure S5D). This loss caused a frameshift and a premature translational termination, leading to a conceptual translation of a peptide of 56 aa, with the first 45 aa being homologous to Myod1 (Additional file 1: Figure S5E). This region contained no functional domains or motifs, suggesting that the peptide is nonfunctional. RT-PCR detection of transcription with primer pair F0/R0 revealed normal expression of *Myod1* in wild-type cells. Whereas no significant transcription was detected in knockout cells (Additional file 1: Figure S5F). Correspondingly, Myod1 was detected in nuclei of wild-type cells but not in knockout cells (Additional file 1: Figure S5G). These confirmed a successful knockout in C2C12 cells. The wild-type cells were termed hereafter as C2C12^*WT*^, and knockout cells were as C2C12^*Myod1*−/−^. Knockout caused cell morphological change. C2C12^*Myod1*−/−^ cells grew long processes, which were not present in C2C12^*WT*^ (Additional file 1: Figure S5H).

**Fig. 8:**
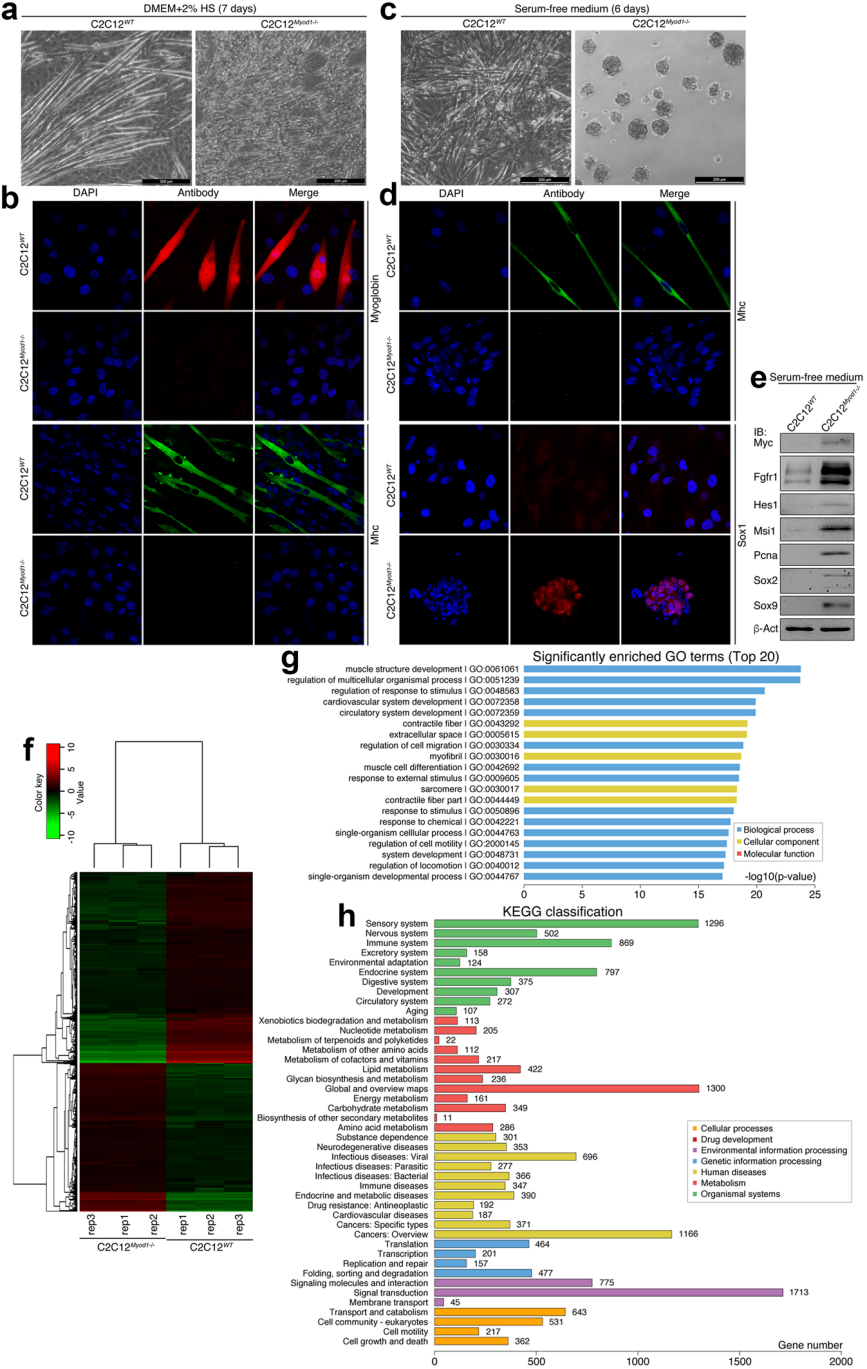
Characterization of the effect of *Myod1* gene knockout (KO) on C2C12 cell identity. **a**, **b** Influence of Myod1 loss on muscle differentiation in C2C12 cells, as viewed by the sharp contrast in morphological changes (**a**) and by IF detection of muscle-specific markers (**b**) in WT and KO cells after 7 days of culture in muscle differentiation medium that contained 2% horse serum (HS). **c**, **d** Evidence for the gain of properties of NSCs/NPCs in KO cells, as revealed by neurosphere formation by KO cells but not by WT cells after 6 days of culture in serum-free medium (**c**). IF detection exhibits also difference in expression of Mhc and Sox1 in these cells (**d**). In **b** and **d**, nuclei were counterstained with DAPI. **e** IB detection of proteins specific for embryonic neural cells in C2C12 WT and KO cells cultured in serum-free medium for 6 days. IB was done with whole cell lysates. β-Act was used as a loading control. **f**–**h** Transcriptomic profiling of WT and KO cells with RNA-sequencing. A RNA-seq heatmap (**f**) shows 3201 DEGs between WT and KO cells, both in triplicate. Red represents upregulated expression, while green means downregulated expression. **g** shows top 20 of enriched GO terms for DEGs, and **h** represents the numbers of DEGs according to KEGG gene classification

C2C12^*WT*^ cells underwent muscle differentiation, as shown by myotube formation in medium with low concentration of serum, typically 2% horse serum; whereas C2C12^*Myod1*−/−^ cells did not (Fig. 8a). The myotubes displayed staining of muscle-specific markers, Myoglobin and Myosin heavy chain (Mhc), which were absent in C2C12^*Myod1*−/−^ cells (Fig. 8b). Therefore, loss of Myod1 led to the loss of muscle differentiation potential. The cells exhibited the ability of muscle differentiation again after introduction of a plasmid coding for MYOD1-eGFP fusion protein, as shown by expression of Mhc and Myoglobin (Additional file 1: Figure S6A), suggesting a successful rescue by compensating the MYOD1 DNA in KO cells. When cultured in serum-free medium, C2C12^*WT*^ cells still formed finer myotube-like structures (Fig. 8c). Intriguingly, C2C12^*Myod1*−/−^ cells formed floating spherical structures, resembling neurospheres (Fig. 8c). IF detection of Mhc confirmed that C2C12^*WT*^ cells indeed formed myotubes (Fig. 8d). But Mhc was not present in the spherical structures (Fig. 8d). Sox1 was not detected in myotube-like structures, but present in spherical structures of C2C12^*Myod1*−/−^ cells (Fig. 8d), an indication of NSCs/NPCs. Immunoblotting (IB) revealed an upregulation of additional neural stemness markers, Fgfr1, Hes1, Msi1, and Sox2/9, the cell cycle protein Pcna and the neural crest regulator Myc in C2C12^*Myod1*−/−^ cells (Fig. 8e). A transcriptome assay showed extensive transcriptional reprogramming after *Myod1* knockout (Fig. 8f), with 1801 genes being downregulated and 1400 genes upregulated (Additional file 8: Table S10). Gene ontology (GO) enrichment analysis indicated that the differentially expressed genes (DEGs) were mostly associated with muscle structure development, followed by other GO terms that are associated with muscle cells and their physiological functions (Fig. 8g). This agrees with the phenotypic change and marker expression assays. Moreover, the largest number of DEGs are neural related genes (sensory plus nervous system) according to KEGG gene classification (Fig. 8H). Interestingly, the human disease corresponding with the biggest number of DEGs is cancer (cancers in general plus cancers of specific types) (Fig. 8h), suggesting a correlation between the neural phenotype and tumorigenicity in knockout cells.

Treatment with retinoic acid (RA), a reagent inducing neuronal differentiation of NSCs/NPCs, did not alter the myotube phenotype (Additional file 1: Figure S6B), but the disaggregated neurosphere cells underwent neuron-like differentiation (Additional file 1: Figure S6B). Mhc was detected in myotubes but not in cells with neuronal morphology (Additional file 1: Figure S6C). In contrast, neuronal markers Map2 and Synapsin-1 were not detected in RA-treated myotubes, but detected in RA-treated C2C12^*Myod1*−/−^ cells (Additional file 1: Figure S6D and E). The results confirmed neuronal differentiation potential of C2C12^*Myod1*−/−^ cells.

**Fig. 9:**
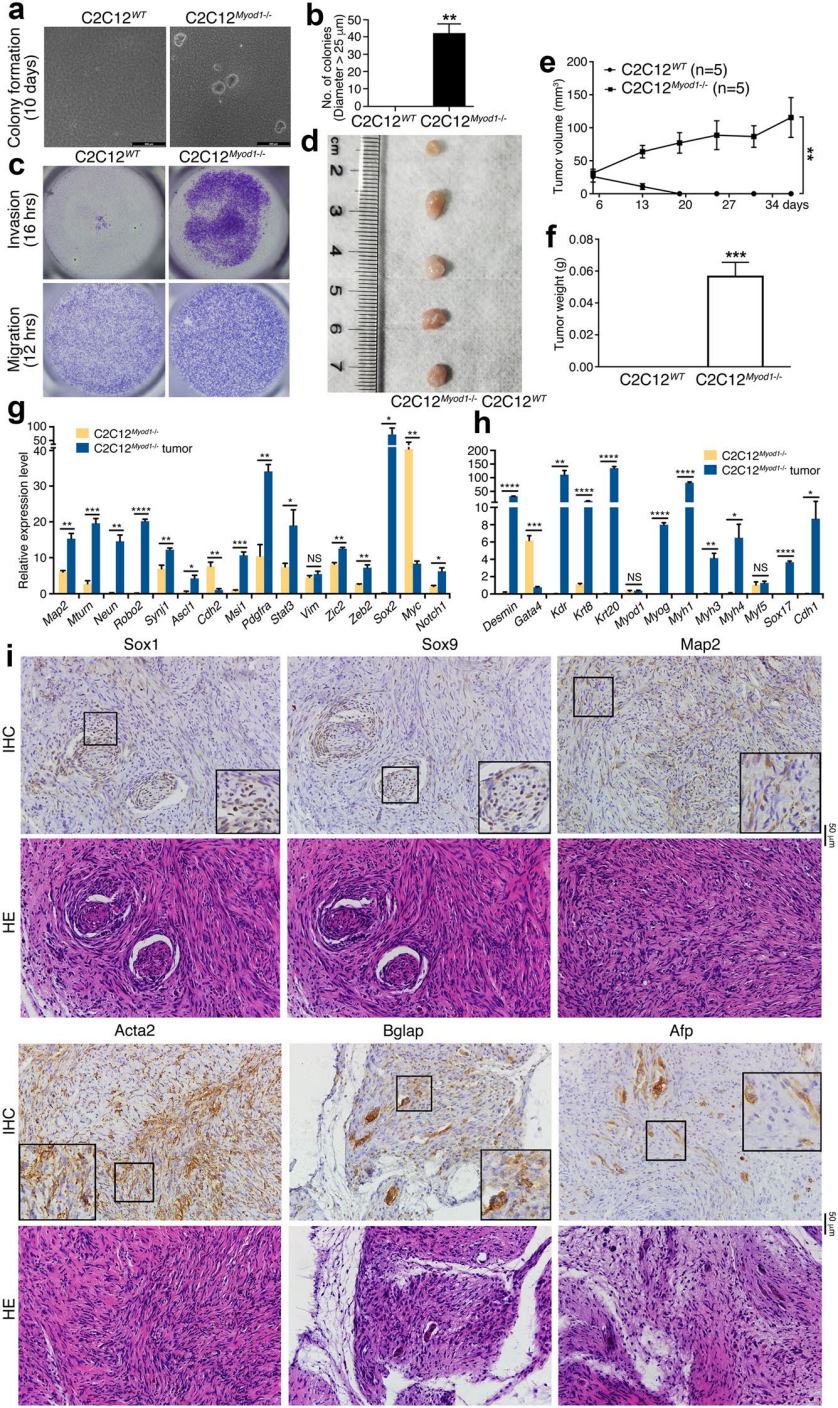
Characterization of tumorigenicity and differentiation potential of WT and *Myod1* gene KO cells. **a**, **b** Soft agar colony formation assay of WT and KO cells during a 10-day period (**a**). Significance in difference of colony formation (**b**) was calculated based on experiments in triplicate using two-tailed Student’s *t*-test. **p < 0.01. Colonies larger than 25 μm in diameter were counted. **c** Difference in invasion and migration of WT and KO cells during time periods as indicated. **d**–**f** Tumor formation of WT and KO cells in nude mice. (D) shows tumor formation in five of five mice injected with KO cells, and **e**, **f** show difference in tumor volume (**e**) and weight (**f**) between WT and KO cells. Significance of difference in tumor volume between two groups of mice was calculated using two-way ANOVA-Bonferroni/Dunn test, and significance of difference in tumor weight was calculated using two-tailed Student’s *t*-test. Data are shown as mean ± SEM. **p < 0.01, ***p < 0.001. **g**–**i** Tissue differentiation in tumor formed by KO cells, as examined by tissue-specific gene expression (**g**, **h**) and marker expression (**i**). **g**, **h** show difference in expression of genes representing neuronal differentiation and neural stemness (**g**), and mesodermal and endodermal tissues (**h**) between KO cells and KO cell xenograft tumors, as detected with RT-qPCR. Significance in expression change was calculated based on experiments in triplicate using two-tailed Student’s *t*-test. Data are shown as mean ± SEM. *p < 0.05, **p < 0.01, ***p < 0.001, ****p < 0.0001. NS: not significant. **i** IHC detection of cell/tissue markers in tumor sections. Below the panels for marker detection are corresponding sections stained with HE. Objective magnification for sections: 20×; insets: 40×

Next we explored whether the gain of NSC/NPC phenotype would mean the gain of tumorigenicity in C2C12^*Myod1*−/−^ cells. In soft agar, C2C12^*WT*^ cells did not form colonies, whereas C2C12^*Myod1*−/−^ cells did (Fig. 9a, b). Moreover, C2C12^*Myod1*−/−^ cells showed stronger abilities in invasion and migration than C2C12^*WT*^ cells (Fig. 9c). These data indicated the gain of malignant features in C2C12^*Myod1*−/−^ cells. Importantly, C2C12^*Myod1*−/−^ cells formed tumor in nude mice, but C2C12^*WT*^ cells did not (Fig. 9d–f; Additional file 1: Table S1). We examined whether there was cell/tissue differentiation in the tumors. RT-qPCR showed a higher expression level of neuronal genes (*Map2*, *Mturn*, *Neun*, *Robo2*, *Synj1*) and neural stemness genes (*Ascl1*, *Msi1*, *Pdgfra*, *Stat3*, *Zic2*, *Zeb2*, *Sox2*, *Notch1*) in tumors than in C2C12^*Myod1*−/−^ cells (Fig. 9g). Moreover, genes representing endodermal and epidermal tissue differentiation *Krt8*, *Krt20*, *Sox17* and *Cdh1*, and genes for mesodermal differentiation *Desmin*, *Kdr*, *Myog*, *Myh1/3/4* were upregulated in tumors (Fig. 9h). IHC revealed expression of Sox1, Sox9, Map2, Acta2, Bglap and Afp in sections, indicating the presence various types of neural and non-neural tissues or cells in tumor (Fig. 9i). There was spindle-cell RMS-like tissue that contained elongated spindle-shaped cell nuclei, for example, the cells in HE-stained sections corresponding with Map2 or Acta2 expressoin (Fig. 9I). This feature is characteristic of spindle cell rhabdomyosarcomas (RMS), which show recurrent MYOD1 p.L122R mutation [31, 32]. This mutation blocks the wild type MYOD1 function [31], causing a loss of function effect that mimics *Myod1* knockout. Actually, MYOD1 was reported as a tumor suppressor. In summary, loss of Myod1 in myoblast cells leads to the loss of their identity, gain of the properties of NSCs/NPCs, tumorigenicity and the potential for re-differentiation.

### Neural stemness is required for tumorigenicity

**Fig. 10:**
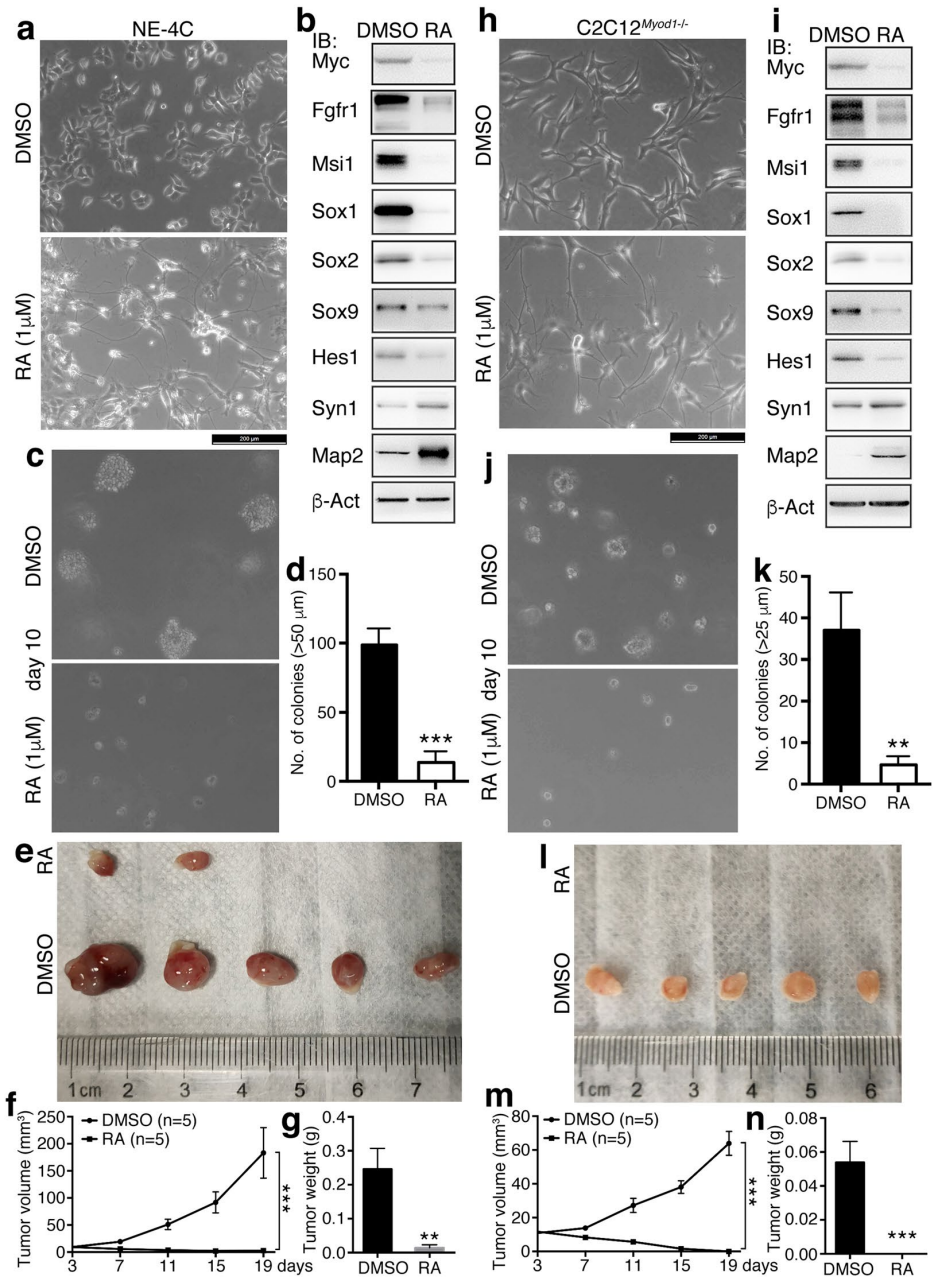
Effect of inhibition of neural stemness in tumorigenic cells on tumorigenicity. **a**–**g** Effect of inhibition of neural stemness in NE-4C cells via RA-induced neuronal differentiation on tumorigenicity. Compared with cells treated with DMSO, NE-4C cells treated RA at 1 μM for 72 h showed dramatic alterations in cell phenotype (**a**), the expression of neural stemness and neuronal proteins as assayed with IB (**b**), and colony formation in soft agar in a 10-day period (**c**, **d**). Significance in difference of colony formation (**d**) was calculated based on experiments in triplicate using two-tailed Student’s *t*-test. ***p < 0.001. Colonies larger than 50 μm in diameter were counted. **e**–**g** shows the difference in tumor formation of cells in nude mice. **e** Shows tumors in five of five mice injected with DMSO or RA treated cells, and **f**, **g** show difference in tumor volume (**f**) and weight (**g**) between the two groups. **h**–**n** Effect of inhibition of neural stemness in KO cells via RA-induced neuronal differentiation on tumorigenicity. Compared with KO cells treated with DMSO, cells treated with RA showed strong difference in cell morphology (**h**), protein expression as assayed with IB (**i**), and colony formation (**j**, **k**). Significance in difference of colony formation (**k**) was calculated based on experiments in triplicate using two-tailed Student’s *t*-test. ***p < 0.001. Colonies larger than 25 μm in diameter were counted. **l**–**n** shows the difference in tumor formation of cells treated with DMSO and RA in nude mice. **l** shows tumors in five of five mice injected with DMSO or RA treated cells, and **m**, **n** show difference in tumor volume (**m**) and weight (**n**) between the two groups. In **f** and **m**, significance of difference in tumor volume between two groups of mice was calculated using two-way ANOVA-Bonferroni/Dunn test. In **g** and **n**, significance of difference in tumor weight was calculated using two-tailed Student’s *t*-test. Data are shown as mean ± SEM. **p < 0.01, ***p < 0.001

The evidence above suggests that the property of neural stemness contributes to cell tumorigenicity. We next tested whether tumorigenic cells changed their tumorigenicity when their neural stemness was reduced. NE-4C cells treated with RA at 1 μM for 72 h showed strongly a neuronal phenotype (Fig. 10a). The treatment caused a significant repression of expression of neural stemness proteins or proteins enriched in embryonic neural cells, Msi1, Sox1/2/9, Hes1, Myc and Fgfr1, which all play promoting roles during tumorigenesis, and upregulation of neuronal proteins Syn1 and Map2 (Fig. 10b), indicating neuronal differentiation and inhibition of neural stemness in NE-4C cells. The differentiated cells showed a dramatic decrease in the ability of colony formation in soft agar (Fig. 10c, d) and compromised tumorigenicity in nude mice (Fig. 10e–g; Additional file 1: Table S1). Similar experiments were carried out for C2C12^*Myod1*−/−^ cells. The cells assumed a neuronal phenotype after treatment with RA (Fig. 10h), and showed a decreased expression of Msi1, Sox1/2/9, Hes1, Myc and Fgfr1 and an increased expression of Syn1 and Map2 (Fig. 10i). This means that neuronal differentiation occurred in the cells accompanied with the reduction of neural stemness property. Likewise, the treated cells displayed decreased ability in colony formation (Fig. 10j, k) and could not form tumors in nude mice, in contrast to vehicle-treated cells (Fig. 10l–n; Additional file 1: Table S1). In addition, we also examined the effect of re-introduction of MYOD1 on tumorigenicity of the C2C12^*Myod1*−/−^ cells. As shown in Additional file 1: Figure S6A, compensation of MYOD1 successfully rescued the KO cells, leading to a muscle cell phenotype in the cells. The rescued cells demonstrated also a reduced capability in colony formation (Additional file 1: Figure S7B and C), with no tumor formation by the rescued cells, in contrast to the control KO cells (Additional file 1: Figure S7E-G and Table S1). These results show that loss of neural stemness via differentiation causes the repression of cell tumorigenicity, indicating that neural stemness is required for tumorigenicity.

### Conservation of neural genes and the their association with cancer

**Fig. 11:**
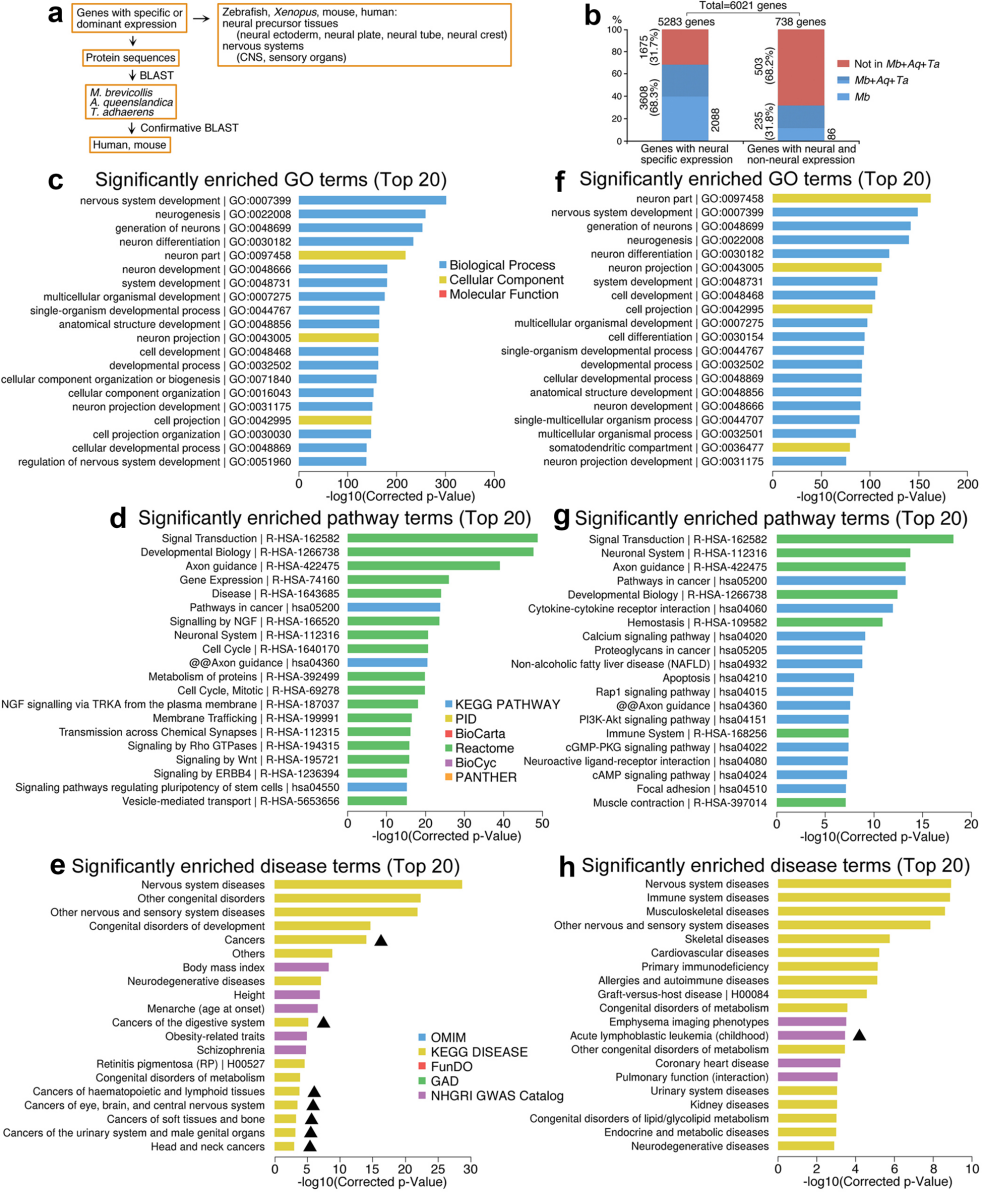
Analysis of neural genes (specific or non-specific), gene conservativeness and their association with diseases. **a** Diagram illustrating the strategy for establishing the relationship between gene expression in vertebrate embryonic tissues and gene conservation. **b** Statistical data showing the numbers (percentages) of genes with neural specific expression or with nonspecific expression that are conserved in *M. brevicollis* (*Mb*), *A. queenslandica* (*Aq*) and/or *T. adhaerens* (*Ta*). **c**–**e** Analysis of association between neural genes and GO (**c**), pathways (**d**) and human diseases (**e**). **f**–**h** Analysis of association between genes with neural but not specific expression and GO (**f**), pathways (**g**) and human diseases (**h**). In **e** and **h**, cancers are highlighted with triangles

Since neural stemness and tumorigenicity are cell properties that are difficult to be interpreted by individual molecular events, e.g. a gene or signaling pathway, we analyzed neural and non-neural genes in general and their association with these cell properties, and explained why neural stemness can contribute to tumorigenicity and differentiation potential. During evolution, ectoderm originated the earliest, followed by endoderm and mesoderm. Moreover, the founders of neural genes increased abruptly first in the last common ancestor of eukaryotes and then at the time of emergence of eumetazoa [33]. We asked whether there was a bias for neural gene evolution during the phase from the unicellular ancestor to metazoan. Three organisms were used for this analysis: *M. brevicollis*, *A. queenslandica* and *T. adhaerens*. *M. brevicollis* is a species of choanoflagellate protists, the closest single-celled relatives of metazoans [34]. *A. queenslandica* represents the oldest surviving metazoan [35] and an evolutionary intermediary between unicellular choanoflagellate protists and eumetazoans, and *T. adhaerens* is the basal eumetazoan species [35, 36]. These organisms shared a last common unicellular ancestor in more than 600 million years ago [34]. By searching tissue expression patterns of genes in typical vertebrate animal models, and comparing protein sequence homology between mammals and these three lower species (Fig. 11a), we found that, among a total of 5283 genes with neural specific expression or at least expression enriched in neural tissues (collectively termed as ‘neural genes’) in *Xenopus*, zebrafish, mouse or human (Additional file 2: Table S4), 3608 genes (68.3%) were found for their founder genes in either one of the three organisms (Fig. 11b; Additional file 2: Table S4). 2088 (39.5%) of neural genes are present in *M. brevicollis* (Fig. 11b; Additional file 2: Table S4). Therefore, most genes for neural cell formation originated already from the starting stage of multicellularity. We also analyzed 738 genes that function in human neuron according to Gene Ontology, but are expressed not specifically or not enriched in neural tissues (Collectively termed as ‘non-specific genes’) (Additional file 3: Table S5). Only 235 (31.8%) genes are present in either one of the three organisms, and 86 (11.6%) genes in *M. brevicollis*. No significant homologs for the remaining genes (68.2%) were identified in these organisms (Fig. 11b; Additional file 3: Table S5). Thus, the genes not specific for neural cells emerged later than neural specific genes.

Neural genes are mostly involved in biological processes for neural development/differentiation and associated with cellular components of neural cells (Fig. 11c). They are involved principally in signal transduction, axon guidance, developmental biology and pathways in cancers (Fig. 11d). These genes are mostly involved, of course, in nervous system diseases, followed by congenital disorders and then by cancers in general and cancers of specific tissues/organs (Fig. 11e). These analyses support an intrinsic link between neural state, embryonic development and cancer.

The 738 non-specific genes are primarily associated with neural development and neuron parts according to GO (Fig. 11f) and with signal transduction, neuronal system, axon guidance and developmental biology according pathway terms (Fig. 11g). They are linked with nervous system diseases and other non-cancer diseases (Fig. 11h). As an additional comparison, we analyzed 7238 genes that are expressed only in non-neural tissues or at least not mainly in neural tissues, or for which the expression patterns are not clear (Additional file 6: Table S8) (Collectively termed as ‘non-neural genes’). The GO and pathway terms enriched for these genes are entirely different from those for neural genes (Additional file 1: Figure S8A and B). These genes are associated predominantly with non-neural, non-cancer and non-congenital diseases (Additional file 1: Figure S8C).

Neuronal genes have over representation of long genes [37, 38]. Long genes are also enriched in cancer pathways [29]. We thus asked whether there was a length bias in the three sets of genes above. Brief calculation showed an average length of 92765 nucleotides (nt) for 11270 transcript variants of 5283 neural genes, an average length of 90154 nt for 1688 transcripts of 738 genes with both neural and non-neural expression, and 42911 nt for 12874 transcripts of 7238 non-neural genes (Additional file 1: Figure S9; Additional file 7: Table S9). Additionally, neural genes have more exons/introns than non-neural genes (Additional file 7: Table S9). Therefore, genes involved in neural development are overall much longer and more complex in gene structure than genes in non-neural cells. In summary, most genes for neural development and differentiation had emerged in the closest species representing the evolution from unicellular organisms to metazoan. Neural genes are tightly connected with cancer and rich in longer genes, whereas non-neural genes are the opposite.

### A neural bias of genes in *M. brevicollis*

**Fig. 12:**
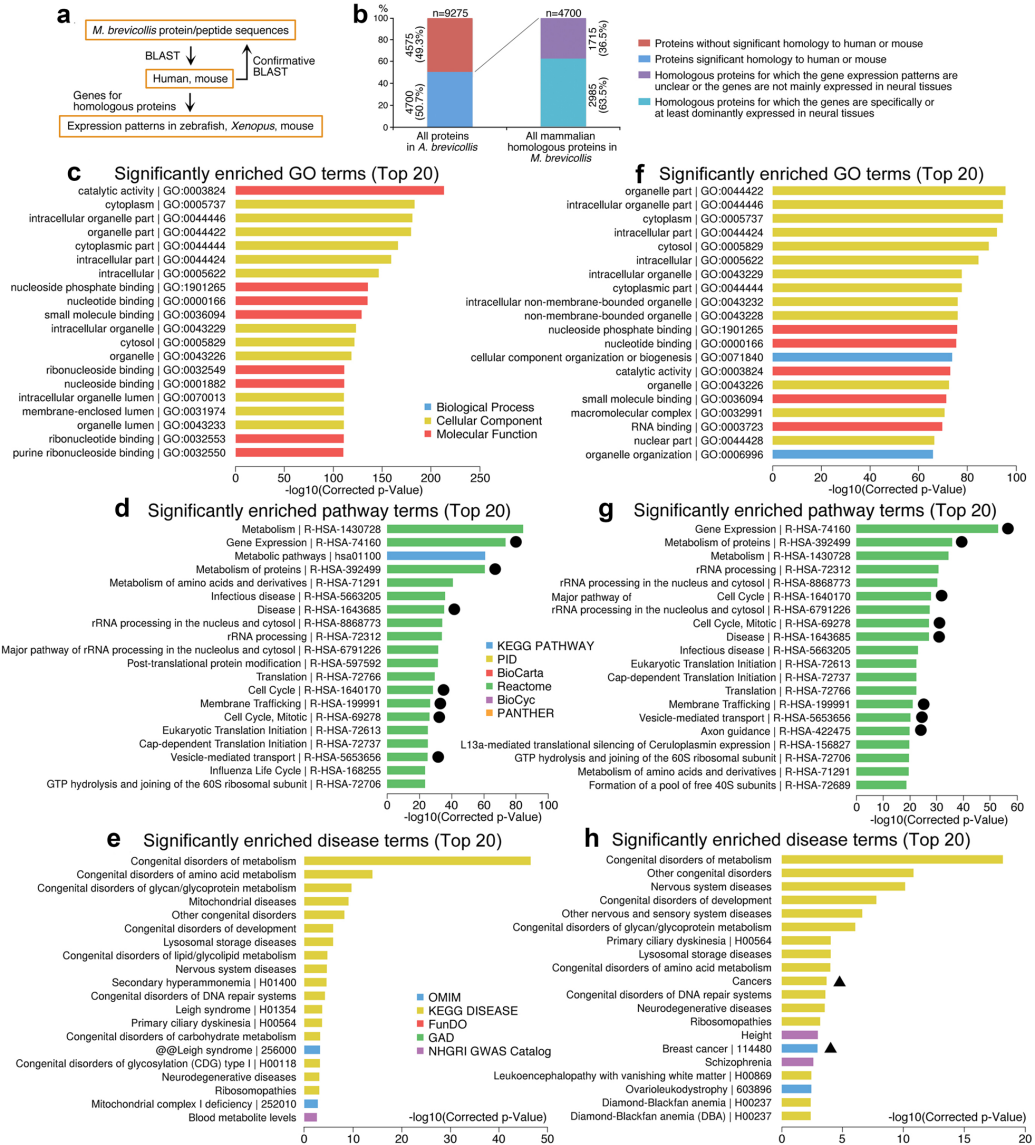
Homology *M. brevicollis* proteins to mammalian proteins, expression patterns of homologous genes, and association analyses. **a** Diagram depicting the strategy for analysis of protein homology between *M. brevicollis* and mammals, and the expression in vertebrate embryonic tissues. **b** Statistical graph showing numbers (percentages) of mammalian homologous proteins in *M. brevicollis*, and the numbers (percentages) of genes for the homologous proteins with neural specific expression in vertebrates. **c**–**e** Association between all mammalian homologous genes in *M. brevicollis* with GO (**c**), pathways (**d**) and human diseases (**e**). **f**–**h** Association between mammalian homologous genes in *M. brevicollis* with neural expression and GO (**f**), pathways (**g**) and human diseases (**h**). Cancers are highlighted with triangles. Circles indicate shared pathway terms between those for mammalian homologous genes in *M. brevicollis* and for neural genes in Fig. 11d

Many neural genes present in *M. brevicollis* let us to analyze whether *M. brevicollis* genes homologous to mammals show expression bias in vertebrates. Among 9275 proteins (Additional file 5: Table S7) encoded by about 9200 genes [34] with *Xenopus*, mouse, or/and human proteins (Fig. 12a), 4700 (50.7%) protein sequences in *M. brevicollis* share significant similarity with those in mouse or/and human, indicating a close relationship between *M. brevicollis* and higher animals (Fig. 12b; Additional file 5: Table S7). Among the 4700 mammalian homologous proteins, the mammalian homologous genes for 2985 proteins, accounting for 63.5%, are neural specific or at least enriched in neural tissues during embryogenesis, whereas spatial expression patterns of the remaining homologous genes are not clear or they are not predominantly expressed in neural tissues (Fig. 12b; Additional file 5: Table S7). Hence, the founder genes in the unicellular organism closest to metazoan might have been biased towards a neural state. This suggests that multicellularity originated from a unicellular neural-biased state.

The 4700 mammalian homologous genes are mainly associated with catalytic activity, subcellular structures and nucleoside/nucleotide/small molecule binding (Fig. 12c). Correspondingly, these genes are correlated with metabolism/metabolic pathways, gene expression, rRNA processing, etc. (Fig. 12d). Logically, these genes are not related with features typical of multicellular animals. For diseases, they are associated with congenital disorders of metabolism and its subcategories (Fig. 12e), in agreement with their functional link with metabolism. The genes are also connected with congenital disorders of development and nervous system diseases. Thus, these genes are mostly related with regulation of cell metabolism, embryonic development and nervous system. There are seven pathway terms (as indicated with circles in Fig. 12d) for *M. brevicollis* genes that are shared with those for neural genes (Fig. 11d). But almost no terms are in common with those for non-specific genes (Fig. 11g) and non-neural genes (Additional file 1: Figure S8B), an additional indication that mammalian homologous genes in *M. brevicollis* are related to neural genes but not the genes for other cell types.

The 2985 mammalian homologous genes with specifically or dominantly neural expression are also mainly associated with subcellular structures, nucleoside/nucleotide/RNA/small molecule binding and catalytic activity (Fig. 12f), and with metabolism, gene expression, rRNA processing and protein translation /metabolism, cell cycle, and axon guidance, etc. (Fig. 12g). This subset of neural genes is mostly associated with various congenital disorders, especially the congenital disorders of metabolism, followed by nervous system diseases and cancers (Fig. 12h), implying an involvement of these genes in development, neural development and cancer. The mammalian homologous genes in *M. brevicollis* are mainly correlated with metabolism, indicating a close relationship in regulatory network for metabolism between higher animals and their unicellular ancestor.

## Discussion

NSCs/NPCs are considered as adult stem/progenitor cell types. That NSCs/NPCs exhibit tumorigenicity might be unexpected. It is not a sheer surprise, however, if we consider that the default fate of embryonic pluripotent cells is neural. Blocking TGFβ signaling in embryonic pluripotent cells enables them to adopt the default fate of NSCs [24–28, 39]. TGFβ is a prime signaling for cell differentiation. It suppresses tumor formation, but exerts a promoting effect on metastasis, immune regulation, etc., of cancer cells. In principle, ESCs without a TGFβ signaling should exhibit stronger tumorigenicity. Indeed, primNSCs derived from ESCs are more tumorigenic than ESCs. PrimNSCs represent the initial neuroectodermal cells during embryonic neural development. NSCs/NPCs derived from later stages of mouse embryos (E9 and E13.5) also exhibited tumorigenicity. In contrast, non-neural cell types, e.g. embryonic fibroblasts and myoblasts, exhibit no tumorigenicity. MSCs are multipotent, though, they exhibit no tumorigenicity [40, 41, present study]. When Myod1 was removed from myoblast cells, or a transcriptional repressor was removed from intestinal stem cells [19], the cells lost their original identity, gain of NSC/NPC property and tumorigenicity, supporting the proposed general principle of tumorigenesis [12]. In either ESCs or somatic cells, blocking pro-differentiation signals leads to the gain of property of neural stemness and enhanced or gain of tumorigenicity. In agreement, loss of pro-differentiation factors or silencing/downregulation of tissue-specific genes is a common feature of tumorigenesis, and many tumor suppressors are pro-differentiation factors, including MYOD1. All these data point to that tumorigenicity is an intrinsic property of neural stemness. This is strengthened by that neural stemness is required for tumorigenicity because loss of neural stemness via forced differentiation leads to the repression of tumorigenicity. Vice versa, cancer cells capable of tumor initiation or tumor initiating cells (TICs) exhibit the properties of neural stemness, such as neurosphere formation and pluripotent differentiation potential (discussed below). Tumorigenesis is a highly dynamic process. Primary tumor cells may have weak neural stemness and mostly still retain the phenotypes of cells-of-origin. Neural stemness may be enhanced gradually during cancer progression and in treatment-failed recurrent tumors.

PrimNSC-derived tumor appeared very similar to ESC-derived teratoma/teratocarcinoma, because the former was also composed of many tissue types derived from all three germ layers. Teratoma/teratocarcinoma formation has been used as a standard approach for the assay of ESC pluripotency. Our data suggest that ESC-derived primNSCs exhibit differentiation potential for different somatic tissues. The pluripotent differentiation potential was also manifested by the contribution of NSCs/NPCs in chimera formation in chick and mouse embryos [24, 42]. In addition, NSCs/NPCs corresponding to later stages of neural development also displayed differentiation potential of diverse tissues of three germ layers. Tissue heterogeneity within or between these tumors reminds of cancer phenotypic heterogeneity. Tumors derived from cancer cell lines also contain different tissue/cell types. Tissue types and tissue phenotypes in these xenograft tumors are generally not as significant as in primNSC-derived tumor. This is probably due to the fact that genes are widely mutated or dysregulated in cancer cells. Therefore, tissue differentiation cannot occur properly as in NSCs, whose genes are intact. Tissue types in each of these tumors may differ; however, a common feature is the presence of immature neuroepithelial cells and differentiated neuron-like cells. Differentiation potential of cancer stem cells (CSCs) has been reported, e.g. those in glioblastoma [16]. Markers representing tissue differentiation are frequently observed in cancers. For example, expression of ACTA2 is universal in cancers, and used as a poor prognostic marker in renal cancer. Other cell/tissue types are also present in different cancer types. For instance, osteoclast-like cells and keratinized epidermal structures were observed in tumors of the pancreas and liver [43], and osteoid formation in breast cancer and melanoma [44, 45], etc. Differentiation potential of CSCs or TICs should also account for cancer types that appear irrelevant with the tissue of origin, e.g. primary osteosarcoma of the breast [45] and the ‘muscle cancer’ (rhabdomyosarcoma) from non-muscle cells [46], for which the mechanisms are unknown.

Tumorigenicity of NSCs resides in that NSCs are the ground state of either embryonic pluripotent cells or somatic cells. The first evidence is the neural default fate of ESCs [24, 25, 39]. Second, somatic cells gain property of NSCs when endogenous factors are blocked [19, 47; present study]. The unique feature of enrichment of longer genes in neural genes enables neural state to be the ground state of differentiation, because longer genes might serve as more flexible scaffolds for regulatory signals required for differentiation of diverse cell types. The neural ground was predestined by the neural biased state of the last common ancestor of unicellular and multicellular organisms. Multicellularity or cell type diversification during evolution was driven by novel genes or regulatory networks, which should inhibit neural genes, thereby repressing neural state to generate non-neural cell types. During ectoderm differentiation, neural fate is achieved by default and epidermal fate is induced [26, 27]. More generally, neural fate is achieved by predetermined ground state, other cells types are all induced. Differentiation will dilute neural ground state and decrease tumorigenicity. During tumorigenesis, one common change is that tissue-specific genes are usually downregulated or silenced in somatic cells. This change leads to de-repression and restoration of neural ground state, a route that needs no inducing signals and is the simplest to follow. The resulting cells will assume a survival strategy that is inherited from primitive ancestors. This agrees with that cancer is driven by ancestral gene regulatory networks, and cancer genes are mostly conserved in unicellular and basal species of multicellular organisms [48–50]. Cancer cell metabolism is characteristic of anaerobic processes, which were the metabolic style of the last common ancestor of unicellular and multicellular organisms in an oxygen-deficient environment in about 600 million years ago. The genes in *M. brevicollis* are mainly associated with cell metabolism. Neural genes could integrate both primitive origin of cancer regulatory networks and anaerobic metabolic processes of cancer cells.

**Fig. 13:**
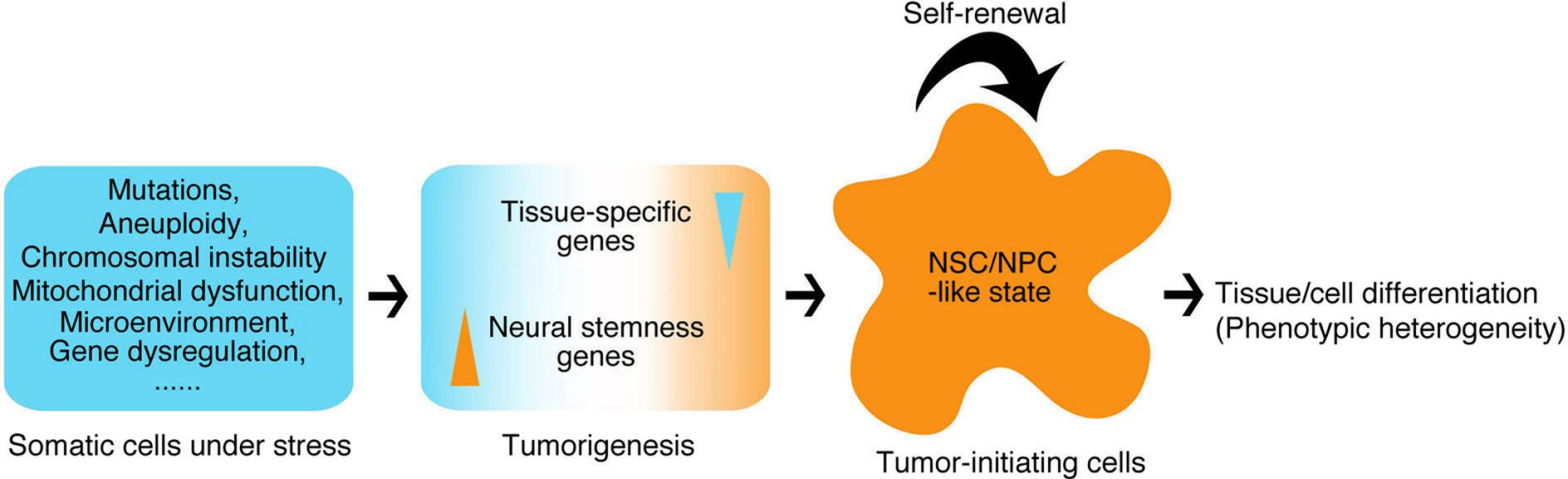
A general model of tumorigenesis. See text for details

To summarize, Fig. 13 describes a general model of tumorigenesis. Somatic cells may suffer various extracellular/intracellular insults, e.g. gene mutations, chromatin instability, microenvironment changes, gene expression changes, etc. If the stresses cause the repression of tissue specific genes or/and upregulation of neural stemness genes, then the result will be the loss of original cell identity, gain of property of neural stemness and tumorigenicity. This is a default route that needs no inducing signals, hence the simplest route to follow. Acquirement of property of neural stemness means the capability of self-renewal and capability of differentiation into different tissue/cell types, which comprise cancer phenotypic heterogeneity.

## Conclusions

By analyzing neural stemness and tumorigenicity of NSCs/NPCs, non-neural embryonic or somatic stem/progenitor cell types, different types of cancer cells, and a gene knockout somatic cell, together with previously reported similarity in gene regulatory networks between embryonic neural cells and cancer cells, we suggest that cell tumorigenicity stems from the property of neural stemness. This is pre-determined by the earlier emergence of neural biased unicellular state, which is the basis for multicellularity and cell type diversification during evolution. Tumorigenesis might be a process of restoration of neural ground state in somatic cells along a default route that is predestined by evolution.

## Supplementary Information

**Additional file 1: Figure S1.** Difference in tumor formation by mESCs and mESC-derived primNSCs in nude mice. (A) Tumors formed by mESCs and primNSCs in nude mice, which were injected at the same number (1×106 cells each mouse). (B-C) Difference in volumes (B) and weight (C) between tumors formed by mESCs and primNSCs. In (B) and (C), data are shown as mean±SEM. Significance of difference in tumor volume between two groups was calculated using two-way ANOVA-Bonferroni/Dunn test, and significance of difference in weight was calculated using two-tailed Student’s t-test. *p<0.05, **p<0.01.** Figure S2**. Tumor formation in nude mice by NE-4C cells injected via tail vein. (A) Tumor formation occurred in different parts of the body, as indicated by arrows. v, ventral view; d, dorsal view. (B) An HE stained section of a tumor dissected from the hind leg, showing the tumor was surrounded by muscle tissue. Objective magnification: 2×.** Figure S3.** Detection of tumorigenicity of MSCs via subcutaneous injection of cells into nude mice. No tumor formation could be observed in the mice in 114 days after injection.** Figure S4.** Detection of tissue origin in xenograft tumors using an antibody specific for human cell nuclei (HuNu). (A) IHC detection of HuNu in sections of tumors derived from different cancer cells. IHC without addition of HuNu antibody was used as negative controls. (B) IHC detection of HuNu in a section of the tumor derived from mouse NE-4C cells. Objective magnification: 4×; insets: 20×.** Figure S5.** Myod1 gene knockout in C2C12 cells using CRISPR/Cas9 and genotyping. (A) Diagram showing mouse Myod1 gene structure, sgRNA, and primer pairs used for genotyping. Numbers indicate position of bases, with the first base of transcription start site being assigned as +1. (B-C) PCR amplification of genomic DNA from a selected clone No. 132 and wild type (WT) C2C12 cells using primer pairs F0/R0 (B) and F1/R1 (C). M: DNA molecular size marker. (D) Sequencing electropherogram showing a deletion of 209 bp in Myod1 gene in the cells of selected clone, which led to a frameshift and premature translational stop. Underlined in the deleted region is the sequence matching sgRNA. (E) Conceptual translation of the truncated Myod1 gene and comparison with the wild type Myod1. (F) RT-PCR detection of Myod1 transcription in wild type and knockout cells using primer pair F0/R0. β-Act was detected as a loading control. RT-: Reverse transcription without transcriptase. M: DNA molecular size marker. (G) IF detection of Myod1 in wild type and knockout cells. Nuclei were counterstained with DAPI. (H) Morphological difference between wild type and knockout cells in C2C12 normal culture medium.** Figure S6.** Rescue of KO phenotype and characterization of neuronal differentiation potential of KO cells. (A) Rescuing effect of electroporation of a plasmid coding for MYOD1-eGFP fusion protein into KO cells. Electroporation of the empty vector pCS2+eGFPmcs was used as a control. Expression of MYOD1 and muscle cell markers was detected with IF. (B) Phenotypic difference of WT and KO cells cultured in serum-free medium for 6 days followed by RA treatment. (C-E) IF assays of muscle-specific (C) and neuron-specific marker (D and E) expression in WT and KO cells, as treated in (B). In (A) and (C-E), nuclei were counterstained with DAPI.** Figure S7.** The effect of re-introduction of MYOD1 on tumorigenicity of C2C12 KO cells. (A) IB detection of forced expression of MYOD1 in C2C12 KO cells. (B and C) The effect of forced expression of MYOD1 on the ability of colony formation in soft agar in a 10-day period. Significance in difference of colony formation (C) was calculated based on experiments in triplicate using two-tailed Student’s t-test. ***p<0.01. Colonies larger than 25 μm in diameter were counted. (E-G) shows the difference in tumor formation between control C2C12 KO cells and cells with forced MYOD1 expression in nude mice. (E) shows tumors in five of five mice injected with control cells but no tumor formation by cells with forced MYOD1 expression, and (F) and (G) show difference in tumor volume (F) and weight (G) between the two groups. In (F), significance of difference in tumor volume between two groups of mice was calculated using two-way ANOVA-Bonferroni/Dunn test. In (G), significance of difference in tumor weight was calculated using two-tailed Student’s t-test. Data are shown as mean±SEM. ***p<0.001.** Figure S8.** Association between the genes that are not expressed in vertebrate neural tissues with GO (A), pathway (B) and human diseases (C). Cancer is highlighted with a triangle.** Figure S9.** Average gene length of three groups of human genes.** Table S1.** Xenograft analysis on different types of cells.** Table S2.** Comparison of tumorigenicity between mESCs and primNSCs by injecting different numbers of cells in mice, as measured 36 days after injection.** Table S3.** Primers for RT-qPCR.

**Additional file 2: Table S4.** 5283 neural specific genes or genes enriched in neural tissues, the evidence for expression patterns, and conservativeness of these genes.

**Additional file 3: Table S5.** 738 genes with neural expression but not specifically or not mainly expressed in neural tissues, the evidence for expression patterns, and conservativeness of these genes.

**Additional file 4: Table S6.** Human neuronal genes retrieved from GO database.

**Additional file 5: Table S7.** Complete list of 9275 proteins in *M. brevicollis*, and homology of these proteins with mammalian proteins, and genes of homologous proteins with neural expression.

**Additional file 6: Table S8.** 7238 genes that are not expressed in neural tissues or for which the expression patterns are unknown.

**Additional file 7: Table S9.** Transcript lengths of 5283 neural genes, 738 non-specific genes and 7238 non-neural genes.

**Additional file 8: Table S10.** 1801 downregulated genes and 1400 upregulated genes in C2C12^*Myod1−/−*^ cells compared with C2C12^*WT*^

## Data Availability

RNA-seq data during the current study are available in the Gene Expression Omnibus (GEO), https://www.ncbi.nlm.nih.gov/geo/, under the accession number GSE137507.
