# Supplementary material for: Neural stemness contributes to cell tumorigenicity
Source: Cell Biosci. 2021 Jan 19;11:21. doi: 10.1186/s13578-021-00531-6 (PMC7814647; doi:10.1186/s13578-021-00531-6)
Supplement: Supplementary file 1 — Additional file 1: Figure S1. Difference in tumor formation by mESCs and mESC-derived primNSCs in nude mice. (A) Tumors formed by mESCs and primNSCs in nude mice, which were injected at the same number (1×10^6^ cells each mouse). (B-C) Difference in volumes (B) and weight (C) between tumors formed by mESCs and primNSCs. In (B) and (C), data are shown as mean±SEM. Significance of difference in tumor volume between two groups was calculated using two-way ANOVA-Bonferroni/Dunn test, and significance of difference in weight was calculated using two-tailed Student’s t-test. *p<0.05, **p<0.01. Figure S2. Tumor formation in nude mice by NE-4C cells injected via tail vein. (A) Tumor formation occurred in different parts of the body, as indicated by arrows. v, ventral view; d, dorsal view. (B) An HE stained section of a tumor dissected from the hind leg, showing the tumor was surrounded by muscle tissue. Objective magnification: 2×. Figure S3. Detection of tumorigenicity of MSCs via subcutaneous injection of cells into nude mice. No tumor formation could be observed in the mice in 114 days after injection. Figure S4. Detection of tissue origin in xenograft tumors using an antibody specific for human cell nuclei (HuNu). (A) IHC detection of HuNu in sections of tumors derived from different cancer cells. IHC without addition of HuNu antibody was used as negative controls. (B) IHC detection of HuNu in a section of the tumor derived from mouse NE-4C cells. Objective magnification: 4×; insets: 20×. Figure S5. Myod1 gene knockout in C2C12 cells using CRISPR/Cas9 and genotyping. (A) Diagram showing mouse Myod1 gene structure, sgRNA, and primer pairs used for genotyping. Numbers indicate position of bases, with the first base of transcription start site being assigned as +1. (B-C) PCR amplification of genomic DNA from a selected clone No. 132 and wild type (WT) C2C12 cells using primer pairs F0/R0 (B) and F1/R1 (C). M: DNA molecular size marker. (D) Sequencing electropherogram showing [file 13578_2021_531_MOESM1_ESM.docx]

**Neural stemness contributes to cell tumorigenicity**

Liyang Xu^1,#^, Min Zhang^1,#^, Lihua Shi^1^, Xiaoli Yang^1^, Lu Chen^1^, Ning Cao^1^, Anhua Lei^1^, and Ying Cao^1,*^

^1^MOE Key Laboratory of Model Animals for Disease Study and Model Animal Research Center of the Medical School, Nanjing University, 12 Xuefu Road, Pukou High-Tech Zone, Nanjing 210061, China

^#^These authors contributed equally to the study

**^*^Correspondence**

Ying Cao, Model Animal Research Center of the Medical School, Nanjing University, 12 Xuefu Road, Pukou High-Tech Zone, Nanjing 210061, China. Email: [caoying@nju.edu.cn](mailto:caoying@nju.edu.cn)

**
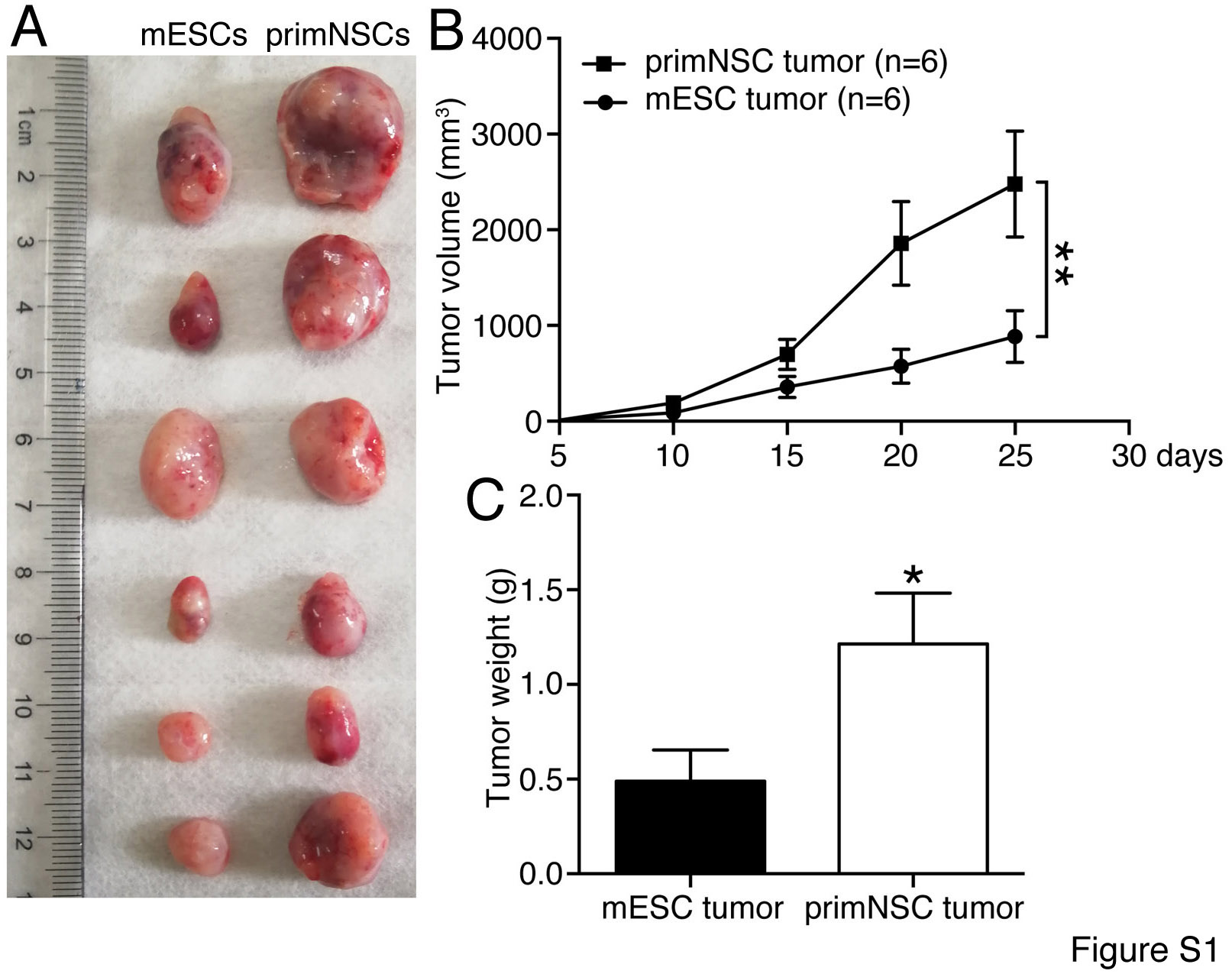
**

**Figure S1** Difference in tumor formation by mESCs and mESC-derived primNSCs in nude mice. (A) Tumors formed by mESCs and primNSCs in nude mice, which were injected at the same number (1×10^6^ cells each mouse). (B-C) Difference in volumes (B) and weight (C) between tumors formed by mESCs and primNSCs. In (B) and (C), data are shown as mean±SEM. Significance of difference in tumor volume between two groups was calculated using two-way ANOVA-Bonferroni/Dunn test, and significance of difference in weight was calculated using two-tailed Student’s *t*-test. *p<0.05, **p<0.01.


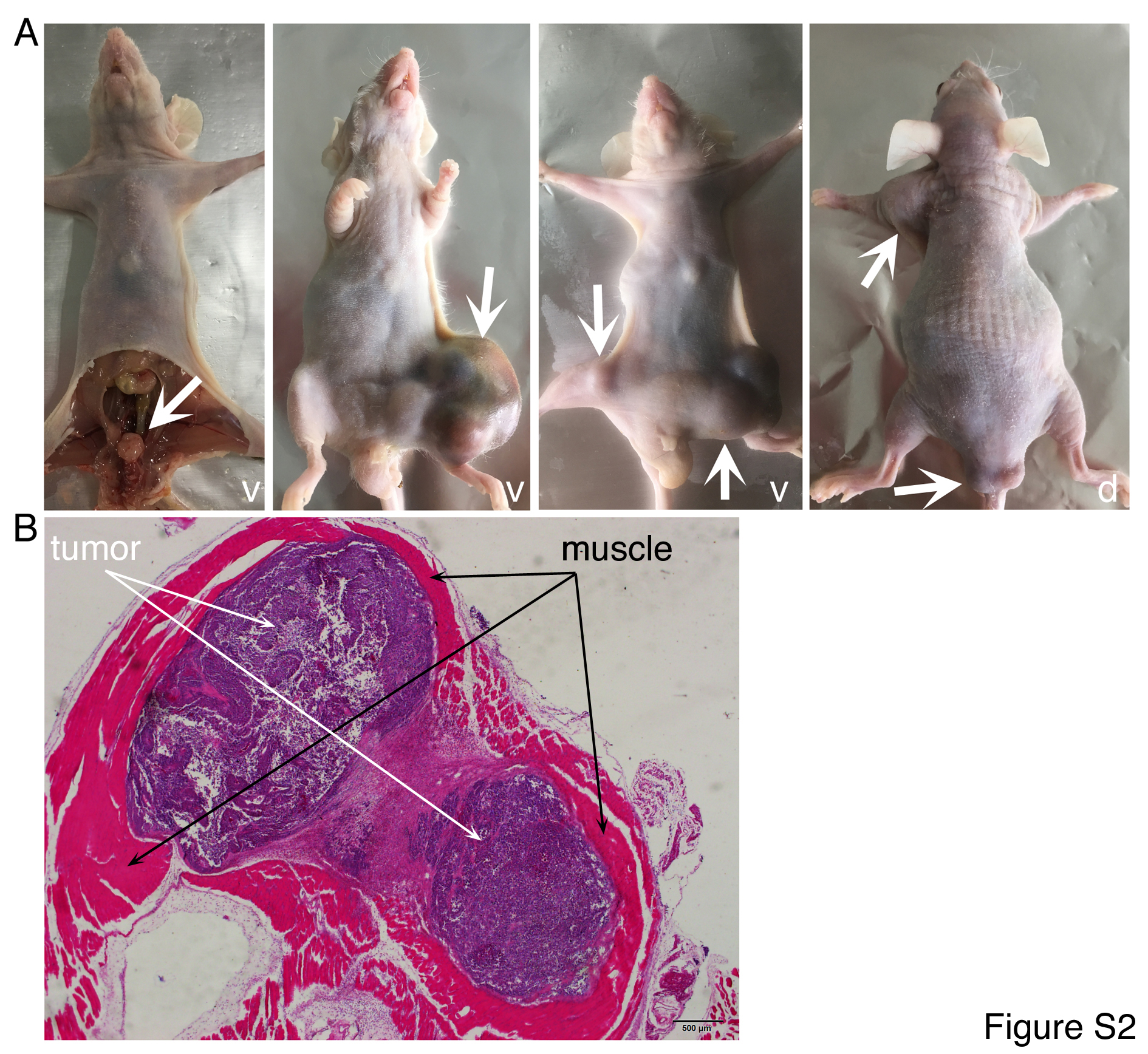


**Figure S2** Tumor formation in nude mice by NE-4C cells injected via tail vein. (A) Tumor formation occurred in different parts of the body, as indicated by arrows. v, ventral view; d, dorsal view. (B) An HE stained section of a tumor dissected from the hind leg, showing the tumor was surrounded by muscle tissue. Objective magnification: 2×.


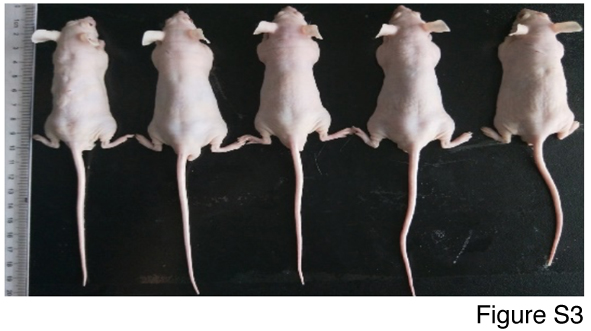


**Figure S3** Detection of tumorigenicity of MSCs via subcutaneous injection of cells into nude mice. No tumor formation could be observed in the mice in 114 days after injection.


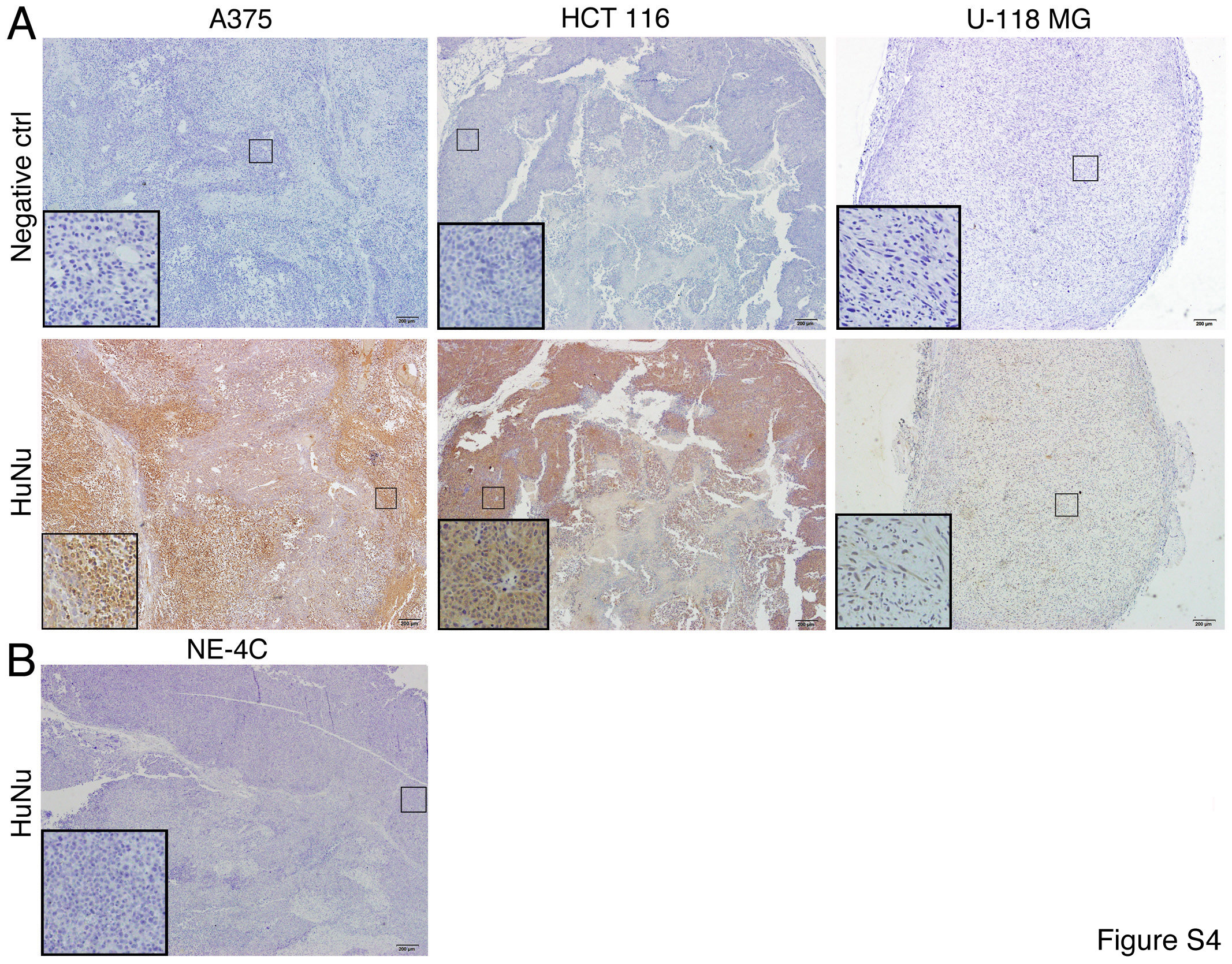


**Figure S4** Detection of tissue origin in xenograft tumors using an antibody specific for human cell nuclei (HuNu). (A) IHC detection of HuNu in sections of tumors derived from different cancer cells. IHC without addition of HuNu antibody was used as negative controls. (B) IHC detection of HuNu in a section of the tumor derived from mouse NE-4C cells. Objective magnification: 4×; insets: 20×.

**
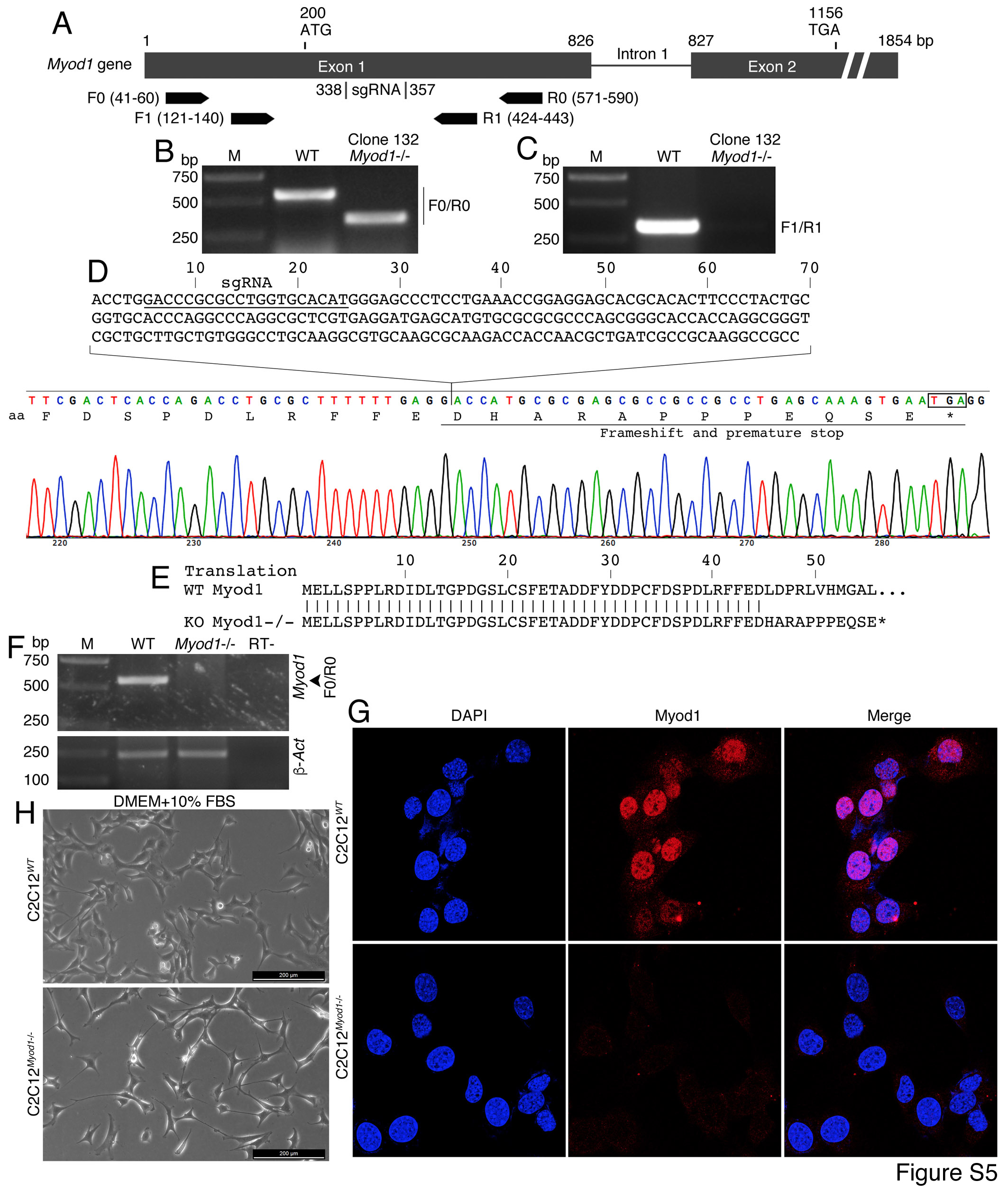
**

**Figure S5** *Myod1* gene knockout in C2C12 cells using CRISPR/Cas9 and genotyping. (A) Diagram showing mouse *Myod1* gene structure, sgRNA, and primer pairs used for genotyping. Numbers indicate position of bases, with the first base of transcription start site being assigned as +1. (B-C) PCR amplification of genomic DNA from a selected clone No. 132 and wild type (WT) C2C12 cells using primer pairs F0/R0 (B) and F1/R1 (C). M: DNA molecular size marker. (D) Sequencing electropherogram showing a deletion of 209 bp in *Myod1* gene in the cells of selected clone, which led to a frameshift and premature translational stop. Underlined in the deleted region is the sequence matching sgRNA. (E) Conceptual translation of the truncated *Myod1* gene and comparison with the wild type Myod1. (F) RT-PCR detection of *Myod1* transcription in wild type and knockout cells using primer pair F0/R0. *β-Act* was detected as a loading control. RT-: Reverse transcription without transcriptase. M: DNA molecular size marker. (G) IF detection of Myod1 in wild type and knockout cells. Nuclei were counterstained with DAPI. (H) Morphological difference between wild type and knockout cells in C2C12 normal culture medium.


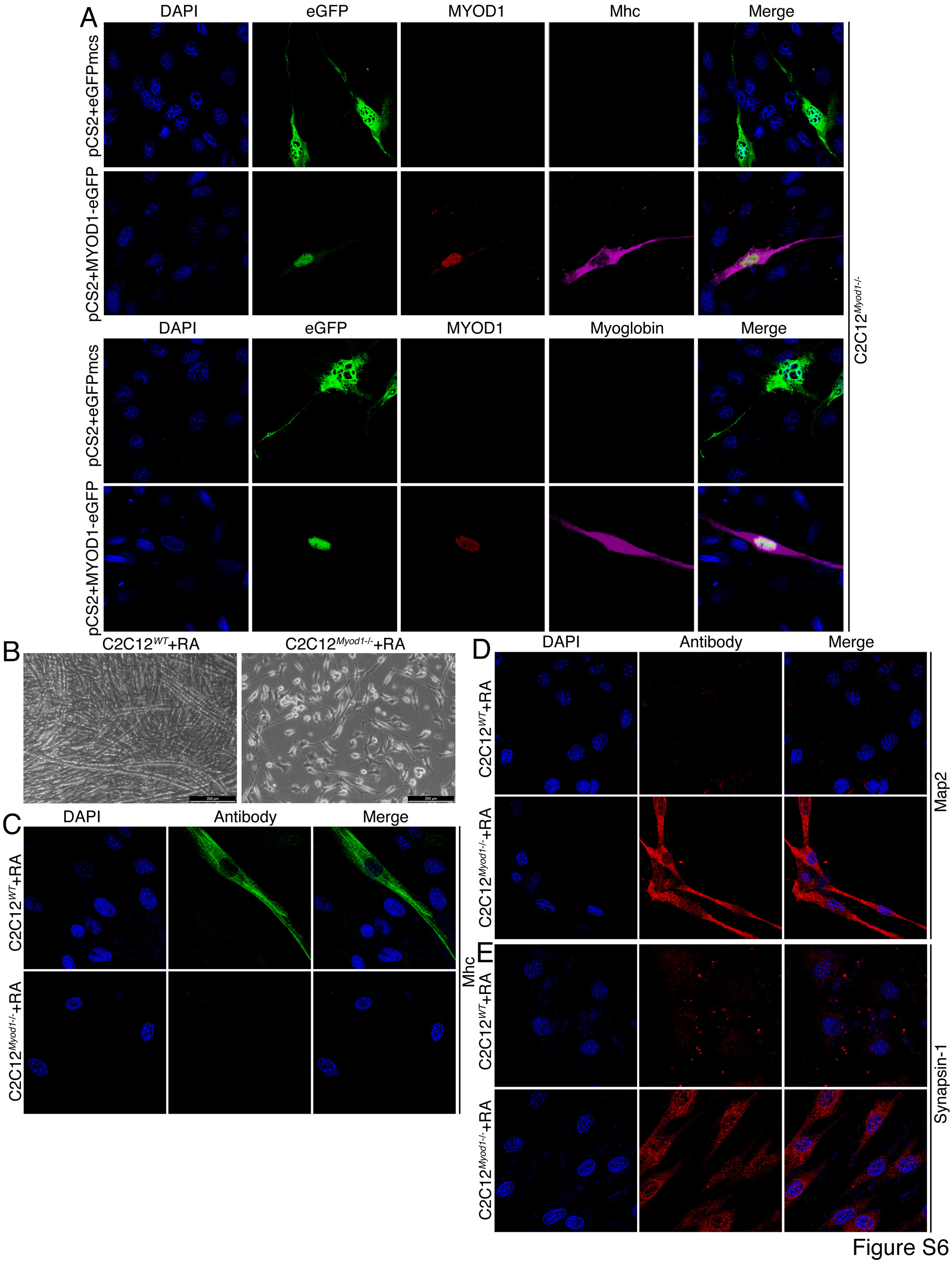


**Figure S6** Rescue of KO phenotype and characterization of neuronal differentiation potential of KO cells. (A) Rescuing effect of electroporation of a plasmid coding for MYOD1-eGFP fusion protein into KO cells. Electroporation of the empty vector pCS2+eGFPmcs was used as a control. Expression of MYOD1 and muscle cell markers was detected with IF. (B) Phenotypic difference of WT and KO cells cultured in serum-free medium for 6 days followed by RA treatment. (C-E) IF assays of muscle-specific (C) and neuron-specific marker (D and E) expression in WT and KO cells, as treated in (B). In (A) and (C-E), nuclei were counterstained with DAPI.


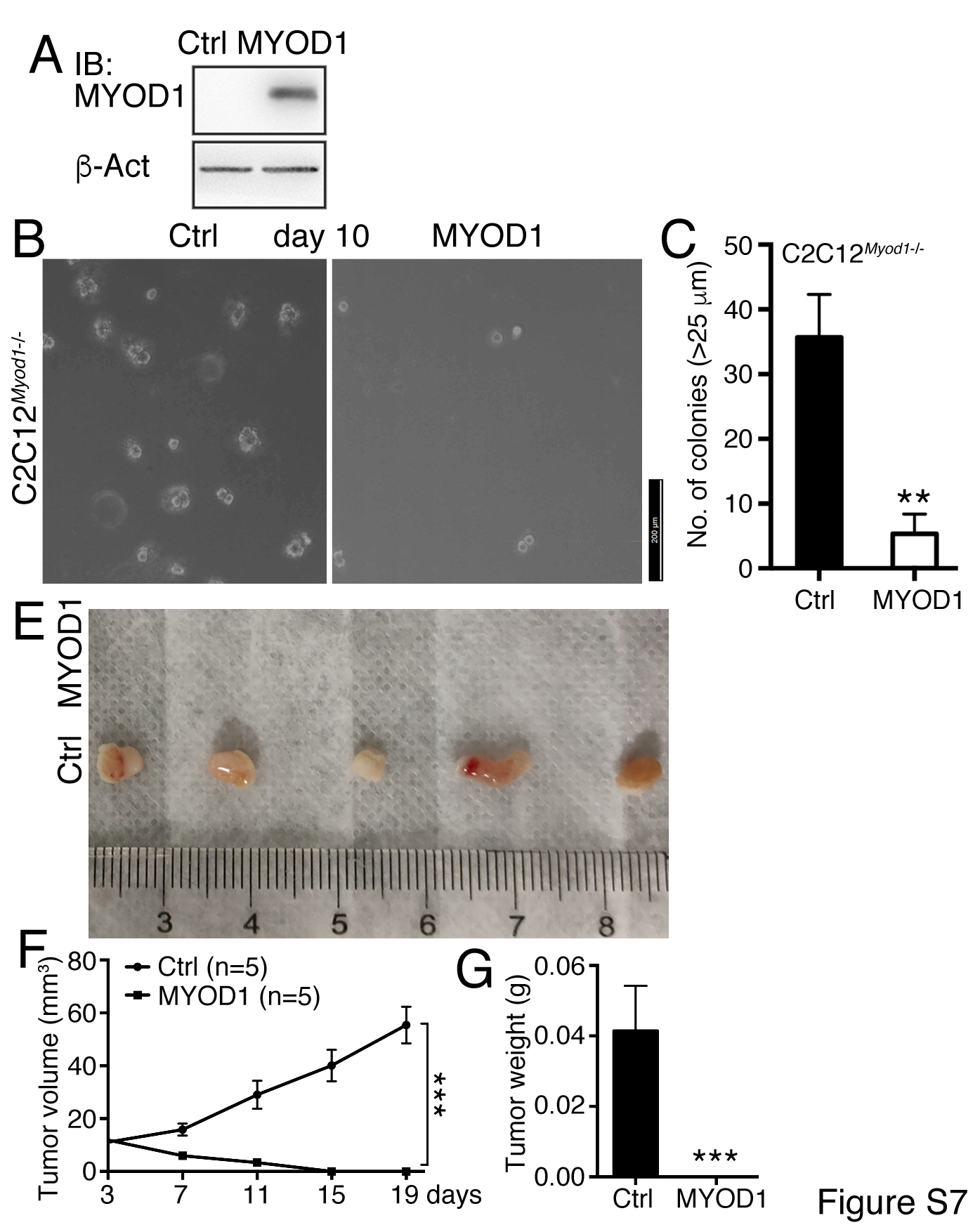


**Figure S7** The effect of re-introduction of MYOD1 on tumorigenicity of C2C12 KO cells. (A) IB detection of forced expression of MYOD1 in C2C12 KO cells. (B and C) The effect of forced expression of MYOD1 on the ability of colony formation in soft agar in a 10-day period. Significance in difference of colony formation (C) was calculated based on experiments in triplicate using two-tailed Student’s *t*-test. ***p<0.01. Colonies larger than 25 μm in diameter were counted. (E-G) shows the difference in tumor formation between control C2C12 KO cells and cells with forced MYOD1 expression in nude mice. (E) shows tumors in five of five mice injected with control cells but no tumor formation by cells with forced MYOD1 expression, and (F) and (G) show difference in tumor volume (F) and weight (G) between the two groups. In (F), significance of difference in tumor volume between two groups of mice was calculated using two-way ANOVA-Bonferroni/Dunn test. In (G), significance of difference in tumor weight was calculated using two-tailed Student’s *t*-test. Data are shown as mean±SEM. ***p<0.001.

**
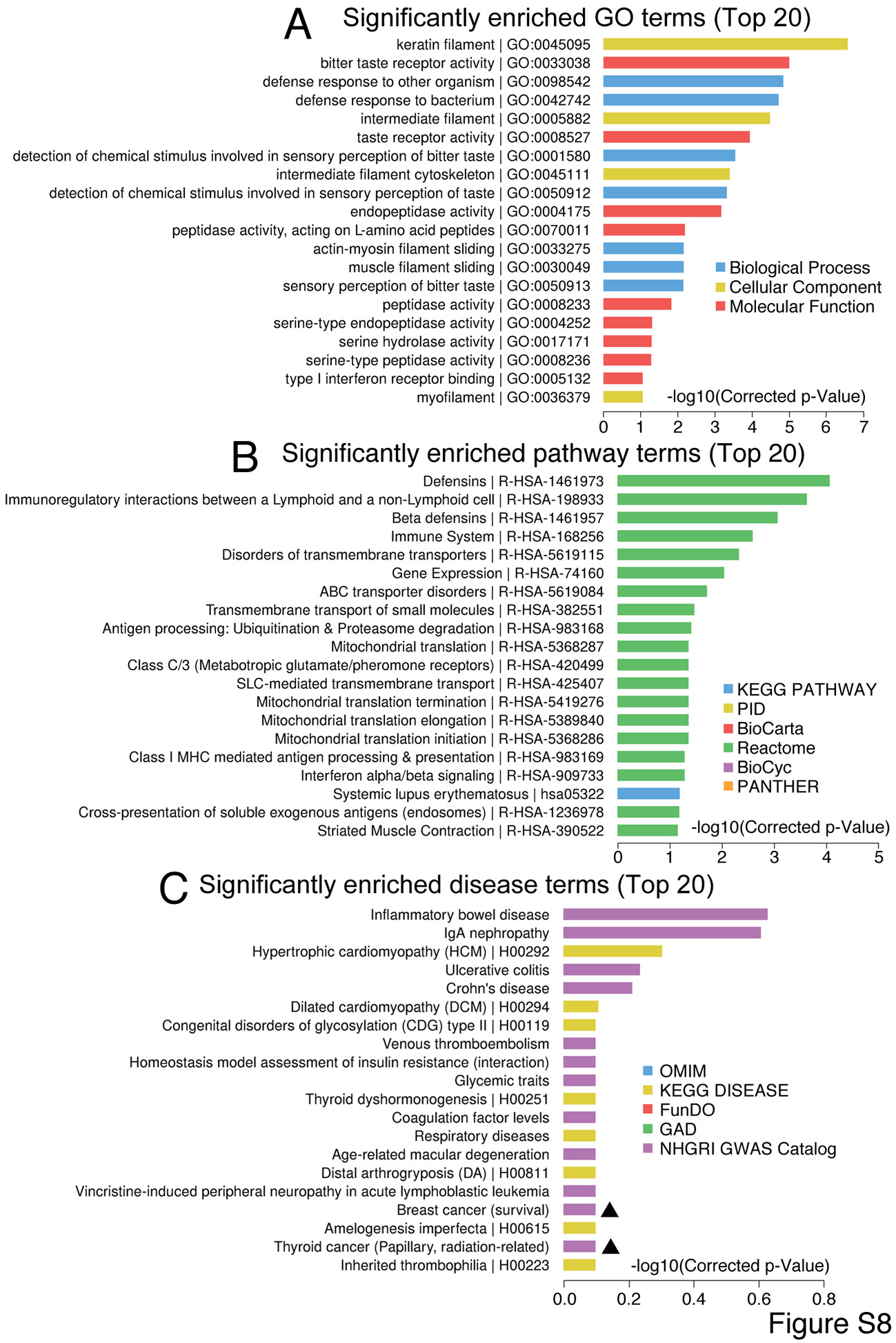
**

**Figure S8** Association between the genes that are not expressed in vertebrate neural tissues with GO (A), pathway (B) and human diseases (C). Cancer is highlighted with a triangle.


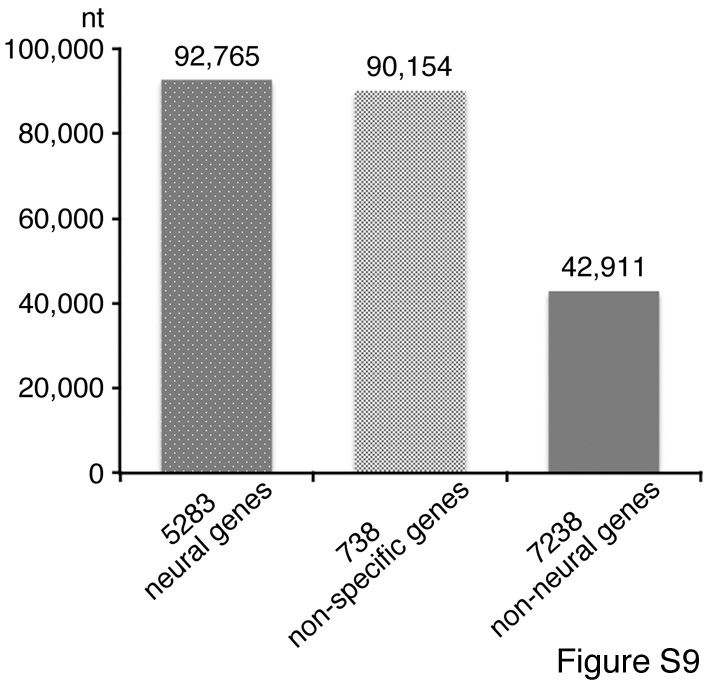


**Figure S9** Average gene length of three groups of human genes.

**Table S1.** Xenograft analysis on different types of cells

| Cell type | Cells injected per mouse | Days after injection | Tumors/Injection |
| --- | --- | --- | --- |
| mESCs | 1×10^6^ | 25 | 6/6 |
| primNSCs derived from mESCs | 1×10^6^ | 25 | 6/6 |
| NE-4C | 2×10^6^ | 35 | 8/8 |
| NE-4C+DMSO | 4×10^6^ | 19 | 5/5 |
| NE-4C+RA | 4×10^6^ | 19 | 2/5 |
| Cortical cells from E13.5 mouse embryos | 5×10^6^ | 47 | 6/8 |
| MEFs from E13.5 mouse embryos | 5×10^6^ | 86 | 0/5 |
| MSC | 2×10^6^ | 114 | 0/5 |
| A375 | 3×10^6^ | 40 | 6/6 |
| HCT 116 | 5×10^6^ | 29 | 6/6 |
| U-118 MG | 4×10^6^ | 34 | 5/5 |
| NCI-H460 | 3×10^6^ | 28 | 6/6 |
| SW480 | 3×10^6^ | 28 | 6/6 |
| U-2OS | 3×10^6^ | 28 | 0/6 |
| HepG2 | 3×10^6^ | 28 | 0/6 |
| C2C12*^WT^* | 5×10^6^ | 44 | 0/5 |
| C2C12*^Myod1-/-^* | 5×10^6^ | 44 | 5/5 |
| C2C12*^Myod1-/-^*+DMSO | 6×10^6^ | 19 | 5/5 |
| C2C12*^Myod1-/-^*+RA | 6×10^6^ | 19 | 0/5 |
| C2C12*^Myod1-/-^* | 6×10^6^ | 19 | 5/5 |
| C2C12*^Myod1-/-^*+MYOD1 | 6×10^6^ | 19 | 0/5 |

**Table S2.** Comparison of tumorigenicity between mESCs and primNSCs by injecting different numbers of cells in mice, as measured 36 days after injection.

| Cells injected per mouse | mESCs (Tumors/Injection) | primNSCs derived from mESCs (Tumors/Injection) |
| --- | --- | --- |
| 1×10^6^ | 10/10 | 10/10 |
| 0.5×10^6^ | 1/12 | 10/12 |
| 1×10^5^ | 0/10 | 4/10 |
| 1×10^4^ | 0/10 | 0/10 |
| 1×10^3^ | 0/10 | 0/10 |

**Table S3.** Primers for RT-qPCR

| Mouse genes | Primers (5’-3’) |
| --- | --- |
| *Acta2* (*alpha-Sma*) | Forward: aatggctctgggctctgtaa Reverse: tctcttgctctgggcttcat |
| *β-Act* | Forward: ccctgaagtaccccattgaa Reverse: cttttcacggttggccttag |
| *Afp* | Forward: atgaagcaagccctgtgaac Reverse: agcttggcacagatccttgt |
| *Ascl1* | Forward: gccaacaagaagatgagcaag Reverse: gaacccgccatagagttcaa |
| *T* | Forward: ctccaacctatgcggacaat Reverse: accattgctcacagaccaga |
| *Cdh1* | Forward: actttggtgtgggtcaggaa Reverse: ttcacatgctcagcgtcttc |
| *Cdh2* | Forward: cggtttcacttgagagcaca Reverse: catacgtcccaggctttgat |
| *Myc* | Forward: acgactccgtacagccctatt Reverse: acgtagcgaccgcaacata |
| *Desmin* | Forward: gtgaagatggccttggatgt Reverse: cgggtctcaatggtcttgat |
| *Foxa2* | Forward: taagcgagctaaagggagca Reverse: gtggttgaaggcgtaatggt |
| *Gata4* | Forward: ggaagcccaagaacctgaat Reverse: tgctgtgcccatagtgagat |
| *Gata6* | Forward: aagatgaatggcctcagcag Reverse: catatagagcccgcaagcat |
| *Gdnf* | Forward: gacttgggtttgggctatga Reverse: aacatgcctggcctactttg |
| *Kdr* | Forward: agctctccgtggatctgaaa Reverse: agatgctccaaggtcaggaa |
| *Krt8* | Forward: tctgggatgcagaacatgag Reverse: tcttcacaaccacagccttg |
| *Krt20* | Forward: tccagacttgaagcccagat Reverse: cagccagcttagcattgtca |
| *Map2* | Forward: agcagccgaagaaacagcta Reverse: aaggtcttgggagggaagaa |
| *Mturn* | Forward: agcgcaggatggacttctac Reverse: ttcatggagggtcttcttgc |
| *Myf5* | Forward: gccatccgctacattgaga Reverse: tgctgtcaaagctgctgttc |
| *Myh1* | Forward: catgtccaaagccaacagtg Reverse: acttggcgttcacagcttct |
| *Myh3* | Forward: tggaagacgaagtgtgagga Reverse: tctcggcaatctgttcagtg |
| *Myh4* | Forward: acattattggctggctggac Reverse: acccttcttcttgccacctt |
| *Myh6* | Forward: gggcattgagtgggagttta Reverse: tgtcatacagcttggccttg |
| *Myod1* | Forward: tcgtgaggatgagcatgtg (Within the deleted region of Myod1 gene in KO cells) Reverse: tctcgaaggcctcattcact (Outside the deleted region) |
| *Myog* | Forward: tccagtacattgagcgccta Reverse: caaatgatctcctgggttgg |
| *Nestin* | Forward: ttccctgatgatccaacctc Reverse: acctctgtggctgcttcttt |
| *Neurod1* | Forward: ctctggagcccttctttgaa Reverse: tgcagggtagtgcatggtaa |
| *NeuN* | Forward: agcagcccaaacgactacat Reverse: tcggtcagcatctgagctagt |
| *Notch1* | Forward: tcgtgctcctgttctttgtg Reverse: agcaccatctgaggcattct |
| *Patched* | Forward: tacgtggaggtggttcatca Reverse: aggcataggcaagcatcagt |
| *Pax5* | Forward: aacttgcccatcaaggtgtc Reverse: ggcgtttgtactcagcgatt |
| *Pax6* | Forward: cacatcaggttccatgttgg Reverse: cataactccgcccattcact |
| *Pdgfra* | Forward: ctcccatccatcaaactggt Reverse: caatctcgacgaagcctttc |
| *Robo2* | Forward: attccgttgtcaggtccaag Reverse: acttttcccacccgattctc |
| *Sox1* | Forward: cacaactcggagatcagcaa Reverse: tccttcttgagcagcgtctt |
| *Sox2* | Forward: gcggagtggaaacttttgtc Reverse: tccgggaagcgtgtacttat |
| *Sox9* | Forward: gctggcaaagttgatctgaag Reverse: gttgggtggcaagtattggt |
| *Sox11* | Forward: cttcatggtgtggtccaaga Reverse: gtcgggataatcagccatgt |
| *Sox17* | Forward: taaaggtgaaaggcgaggtg Reverse: tagctctgcgttgtgcagat |
| *Stat3* | Forward: caaaaccctcaagagccaag Reverse: ttctgcacgtactccattgc |
| *Synj1* | Forward: gagctgaatgctgggaacat Reverse: gcgtgctgtggtctgataaa |
| *Tubb3* | Forward: ttctggtggacttggaacct Reverse: actctttccgcacgacatct |
| *Vim* | Forward: gaccttgaacggaaagtgga Reverse: agccacgctttcatactgct |
| *Zeb2* | Forward: gggacagatcagcaccaaat Reverse: gcagtttgggcattcgtaag |
| *Zic1* | Forward: tggagccttcttccgctat Reverse: actcctcccagaagcagatgt |
| *Zic2* | Forward: tccgagaacctcaagatcca Reverse: tgggatgcgtgtaggactt |
|  |  |
| Human genes | Primers (5’-3’) |
| *ACP5* | Forward: ttccaggagacctttgagga  Reverse: tgtgggatcttgaagtgcag |
| *ACTA2* (*alpha-SMA*) | Forward: ctgttccagccatccttcat Reverse: ccgtgatctccttctgcatt |
| *β-ACT* | Forward: agaaaatctggcaccacacc Reverse: tagcacagcctggatagcaa |
| *AFP* | Forward: agcttggtggtggatgaaac Reverse: tctgcaatgacagcctcaag |
| *ASCL1* | Forward: gtgcgaatggactttggaag Reverse: gggttggttgactgttttcg |
| *BDNF* | Forward: tgttggatgaggaccagaaag Reverse: cctcatggacatgtttgcag |
| *BGLAP* | Forward: cctttgtgtccaagcagga  Reverse: tgaaagccgatgtggtcag |
| *T* | Forward: aagtacgtgaacggggaatg Reverse: tgagcttgttggtgagcttg |
| *CALCR* | Forward: ggcctgcaactatttctgga  Reverse: aatgggtttccacactcagc |
| *CDH1* | Forward: tggacagggaggattttgag Reverse: acctgaggctttggattcct |
| *CDH2* | Forward: ccatcactcggcttaatggt Reverse: acccacaatcctgtccacat |
| *MYC* | Forward: tcaagaggcgaacacacaac Reverse: atgagcttttgctcctctgc |
| *CTSK* | Forward: tgtggtgagctttgctctgt  Reverse: gcctcaaggttatggatgga |
| *DESMIN* | Forward: tatgagaccatcgcggcta Reverse: ggaatcgttagtgcccttca |
| *FOXA2* | Forward: cccacaaaatggacctcaag Reverse: ataatgggccgggagtaca |
| *FGF5* | Forward: agtcaatggatcccacgaag Reverse: cttgaaaacgctccctgaac |
| *GAPDH* | Forward: tcaagaaggtggtgaagcag Reverse: tgacaaagtggtcgttgagg |
| *GATA4* | Forward: taagacaccagcagctccttc Reverse: atcgcactgactgagaacgtc |
| *GATA6* | Forward: gtgtgcaatgcttgtggact Reverse: agttggagtcatgggaatgg |
| *GFAP* | Forward: aggaagattgagtcgctgga Reverse: atactgcgtgcggatctctt |
| *HNF1A* | Forward: cctcaaagagctggagaacct Reverse: ttgttgaggtgttgggacag |
| *HNF4A* | Forward: gtgtccatacgcatccttga Reverse: tactggcggtcgttgatgta |
| *KDR* | Forward: atccctgtggatctgaaacg Reverse: agatgctccaaggtcaggaa |
| *KRT8* | Forward: caagaaggatgtggatgaagc Reverse: caatgatgctgtccatgtcc |
| *KRT20* | Forward: atgaagtcatggcccagaag Reverse: ttcatgctgagatgggactg |
| *NCAM1* | Forward: cagatgggagaggatggaaa Reverse: ttcagcatgacgtggtcact |
| *NEUN* | Forward: gagaagctgaatgggacgat Reverse: aaccccgtcactgcatagaa |
| *MAP2* | Forward: cagggaggaatttgtggaga Reverse: atggtctcctttccacctca |
| *MSI1* | Forward: accaagagatccaggggttt Reverse: tcgttcgagtcaccatcttg |
| *MTURN* | Forward: acgcaggatggatttctacg Reverse: acttcgtggagcgtcttctt |
| *OCT4* | Forward: tgagaggcaacctggagaat Reverse: cagcagcctcaaaatcctct |
| *PAX6* | Forward: aagggccaaatggagaagag Reverse: gccagatgtgaaggaggaaa |
| *PDGFRA* | Forward: ggccccatttacatcatcac Reverse: catagctccgtgtgctttca |
| *ROBO2* | Forward: acccttccagcaagtgcata Reverse: gttgatcgctctgaccatga |
| *SOX1* | Forward: aagtcaaaacgaggcgagag Reverse: aagtgcttggacctgcctta |
| *SOX2* | Forward: catcacccacagcaaatgac Reverse: cctccccaggttttctctgta |
| *SOX9* | Forward: ccgaagaaagagaggaccaa Reverse: gcgcttggataggtcatgtt |
| *SYN1* | Forward: aatactggctctgcgatgct Reverse: tgtcttcatcctggtggtca |
| *SYNJ1* | Forward: tggcttctgaacagttggtg Reverse: agcaaaggctggttgtatgg |
| *SYT1* | Forward: gccatagtcgcagtcctttt Reverse: tgagggcctgatctttcatc |
| *TUBB3* | Forward: gtgcggaaggagtgtgaaa  Reverse: acgacgctgaaggtgttcat |
| *VIM* | Forward: tgttgacaatgcgtctctgg Reverse: ggcttggaaacatccacatc |
| *ZEB2* | Forward: caccaaatgctaacccaagg Reverse: tgtgcgaactgtaggaacca |
| *ZIC1* | Forward: acaaaaggacgcacacagg Reverse: ggattcgtggaccttcatgt |
| *ZIC2* | Forward: gtacggccccatgaatatga Reverse: cttgggattgctcagttgct |
